# How a school holiday led to persistent COVID-19 outbreaks in Europe

**DOI:** 10.1038/s41598-021-03927-z

**Published:** 2021-12-22

**Authors:** Björn Thor Arnarson

**Affiliations:** Department of Economics, University of Copenhagen, Copenhagen, Denmark

**Keywords:** Infectious diseases, Risk factors

## Abstract

This paper investigates the role of large outbreaks on the persistence of Covid-19 over time. Using data from 650 European regions in 14 countries, I first show that winter school holidays in late February/early March 2020 (weeks 8, 9 and 10) led to large regional outbreaks of Covid-19 in the spring with the spread being 60% and up-to over 90% higher compared to regions with earlier school holidays. While the impact of these initial large outbreaks fades away over the summer months, it systematically reappears from the fall as regions with school holidays in weeks 8, 9 and 10 had 30–70% higher spread. This suggests that following a large outbreak, there is a strong element of underlying (latent) regional persistence of Covid-19. The strong degree of persistence highlights the *long-term* benefits of effective (initial) containment policies, as once *a* large outbreak has occurred, Covid-19 persists. This result emphasizes the need for vaccinations against Covid-19 in regions that have recently experienced large outbreaks but are well below herd-immunity, to avoid a new surge of cases.

## Introduction

**Figure 1:**
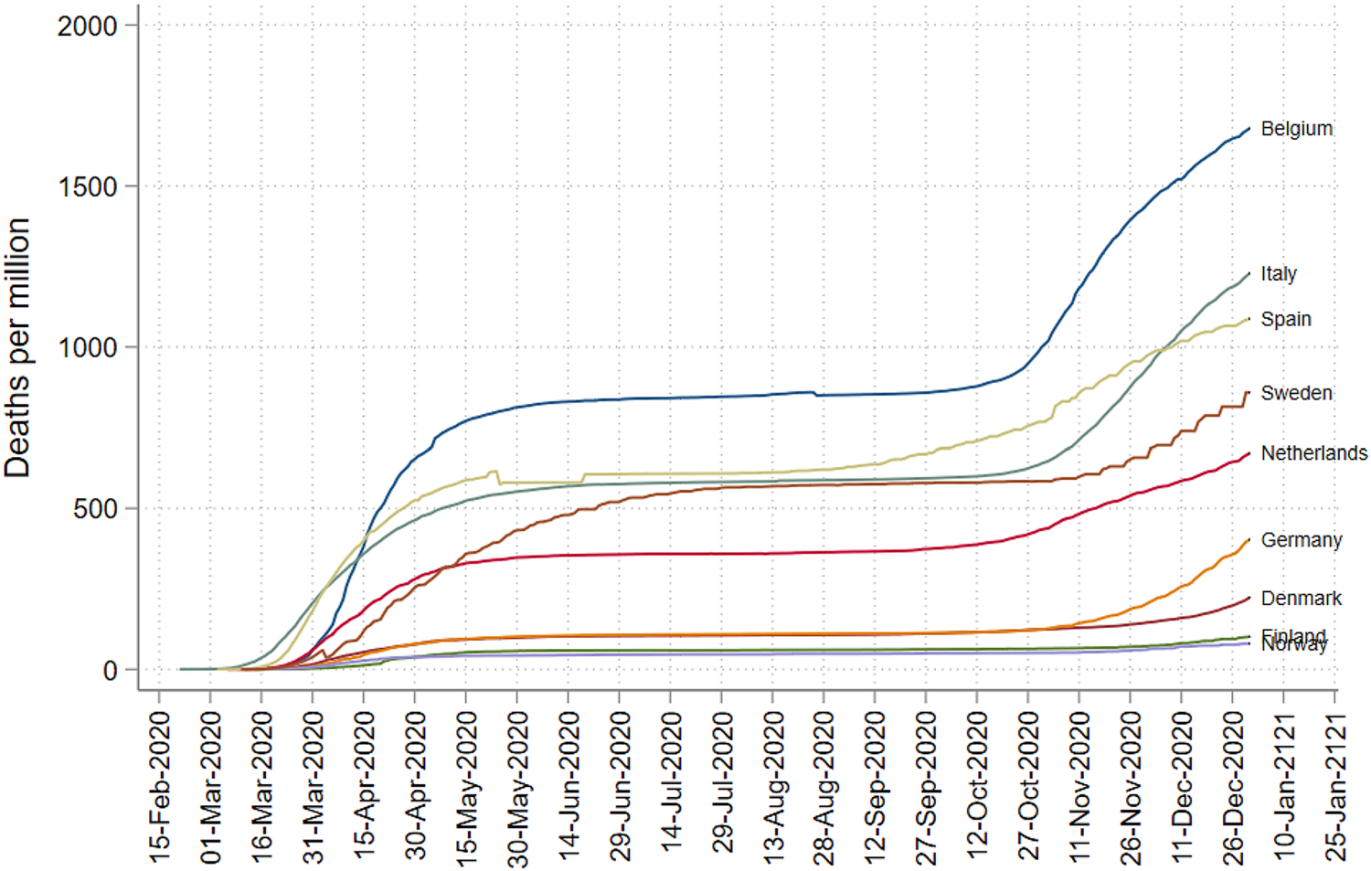
The cumulative number of confirmed Covid-19 deaths per capita in Europe (selected countries). Source: Figure created by the author using data from Dong et al.^1^.

In early March 2020, Europe became the center of the Covid-19 pandemic, with the number of cases and deaths increasing exponentially. On March 11th the WHO declared Covid-19 a pandemic and containment measures intensified across Europe. Notable differences could however be seen both within and between similar countries. From Fig. 1 we can see how countries that had a relatively high number of deaths per capita in the spring are relatively hard hit from the fall. Hence, the patterns persist even after the summer holiday months, when the spread of Covid-19 appeared minimal.

In this paper, I contribute to the understanding of these patterns in the data. The main contributions are twofold. First, as Covid-19 was only found in a limited number of places in Europe in mid-February, human transportation was needed to distribute the virus to new places. I show that the clustered school holidays during this critical period played a large role in the initial distribution of Covid-19. Secondly, I show how the impact of large initial outbreaks still persists in the fall/winter of 2020 even after various efforts to contain the spread of Covid-19. Hence, areas with high initial exposure (school holiday in week number 9, 10 or 8) are consistently relatively worse hit in the fall and early winter 2020.

## Background

First cases of Covid-19 were identified in Europe in January 2020 and only sporadic cases reported until middle of February. From the WHO Covid-19 situation report on February 21st, only 47 cases had been confirmed in Europe and 1200 outside of China, over half of which were linked to the Diamond Princess cruse-ship. The situation escalated rapidly in Europe from this point, and on March 13th the Director-General of the World Health Organization noted that “*Europe has now become the epicenter of the pandemic, with more reported cases and deaths than the rest of the world combined, apart from China.*” Hence, in the short time-span from the 21st of February until the 13th of March, Covid-19 took hold and spread uncontrolled throughout Europe.

During this pivotal period, in late February and early March, many European countries had school holidays. The specific naming and purpose varies from e.g. winter sport-holidays, half-term holiday, carnival/crocus and some even had early spring breaks. The generic term *school holiday* is used in this paper to describe all school holidays at the primary and secondary education level in the period of interest (January to March 2020).

**Figure 2:**
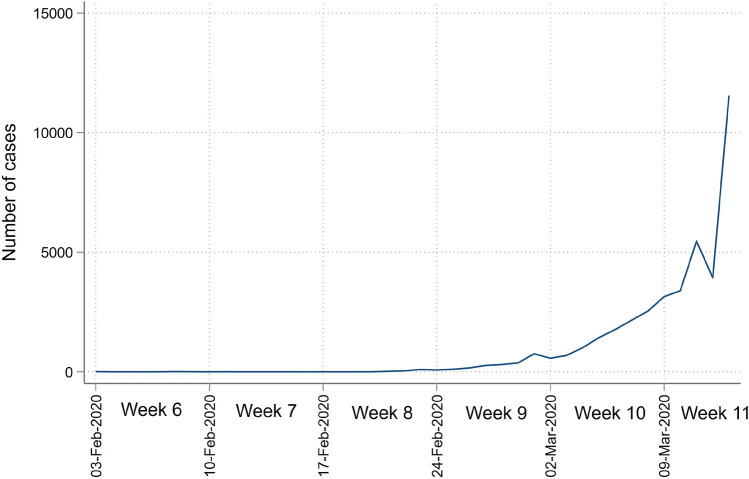
The number of new confirmed cases of Covid-19 in Europe in late February/early March 2020. Source: Figure created by the author using data from Hasell et al.^2^.

Before proceeding further, it is useful to create a timeline for the spread of Covid-19 in Europe to pinpoint which weeks were, at the time, *thought* to have been safe for travel and which weeks, ex-post, are most likely related to high exposure. From Fig. 2 we can see that only a handful of confirmed cases were being reported in weeks 6 and 7 and only after February 20 did the number of (confirmed) cases start increasing rapidly. We can therefore broadly generalize the likely impact of the school holiday by week.

- **Week 7 (10–16 February or earlier):** A school holiday during or before week 7 is not likely to spark a large outbreak, as the spread of Covid-19 was sporadic/localized, with the number of confirmed daily cases below 10 in Europe.
- **Week 8 (17–23 February):** By the end of week 8 the number of reported cases was increasing, suggestive of local transmission. Number of daily cases reached around 100.
- **Week 9 (24 February–1 March):** During this week, the number of cases increased rapidly, with daily cases above 750 by the end of the week.
- **Week 10 (2–8 March):** During week 10 the exponential spread continued with over 2000 daily cases at the end of the week. From late week 10 and start of week 11 the severity of the pandemic becomes more tangible through, for example several, large (global) stock market declines (March 9th, 11th, 12th and 16th), WHO declared Covid-19 a pandemic and the US travel ban on many European countries (both on March 11th).

It is important to stress that the number of cases during these weeks is now known to have been underestimated, as most cases were undetected^3^. However, the numbers give a good picture of how the spread of Covid-19 was *thought* to have been at the time. Public awareness of the seriousness prior/during travel was low until late week 10 (early week 11). Hence, a traveler in week 6, 7, 8 or 9 would only see a limited number of confirmed cases prior to travel (in weeks 5, 6, 7 and 8) but the likelihood of being exposed to Covid-19 would be vastly different. Unaware individuals traveling in the high-exposure week 9 are therefore likely to have provided human transport of Covid-19 to their local area. During the short time window around week 9, the risk of being exposed to Covid-19 during travel was high, while the *perceived* risk was low. As the awareness of the risks was still rather low on return, these individuals are in addition likely to have started their normal lives before the seriousness of the spread was apparent across Europe, amplifying the local geographic exposure. A similar argument applies for week 8, but from a lower base. While the spread was higher in week 10, than 9, the seriousness was more noticeable and hence less clear if week 10 travelers are more or less likely to have brought Covid-19 to their home region.

Contact tracing data from Denmark, Sweden and Norway, provides evidence that the surge of cases in March 2020 was mostly related to traditional winter holiday destinations such as Austria (1150 cases) and Italy^4^. Similar evidence is provided from analyzing haplotypes in Denmark and Iceland^5,6^). Notable as well, that during extensive contact tracing in Iceland in March/April 2020, only 2 of the 200 cases could be traced to foreigners/tourists. Hence, the bulk of the initial spread could be attributed to locals returning from abroad and related subsequent spread^7^.

School holidays in Europe vary considerably, both within and across countries. Some countries lump the break in a single week (Belgium in week 9) others stagger the break over multiple weeks (e.g. Netherlands, Sweden, Germany, Slovakia, Denmark). Given the timeline constructed above, we expect the likelihood of a regional outbreak to vary substantially depending on the week of the school holidays. Regions with a school holiday *prior or during* week 7 are not likely to experience outbreaks, but as the pandemic intensifies over time, the likelihood increases of a large clustered outbreak on return from travel. The school holidays may therefore lead to both within and cross-country variation in the initial spread of Covid-19. Since school holidays are generally either region-week or country-week specific, they may lead to multiple simultaneous introductions of Covid-19. The geographic and week wise *clustering* of school holidays increases therefore the likelihood of multiple simultaneous independent cases being introduced into a sub-national area upon return from travel during the school holiday. This clustering is significant since^8^ find that once at least four (ten) *independent* cases of Covid-19 have been introduced into a new location, there is over 50% (90%) chance that a *large* outbreak will occur. This also underscores why school holidays are potentially more significant for the initial exposure than business travel, which tends to be less clustered by both geography and time.

To isolate the impact of the school holidays, we use only data from countries that fulfill the following criteria: (1) the regional variation in school holidays is clear and clustered in both time and space, and (2) the domestic spread did not take off due to other events during the holidays. See Appendix B for more information.

**Figure 3:**
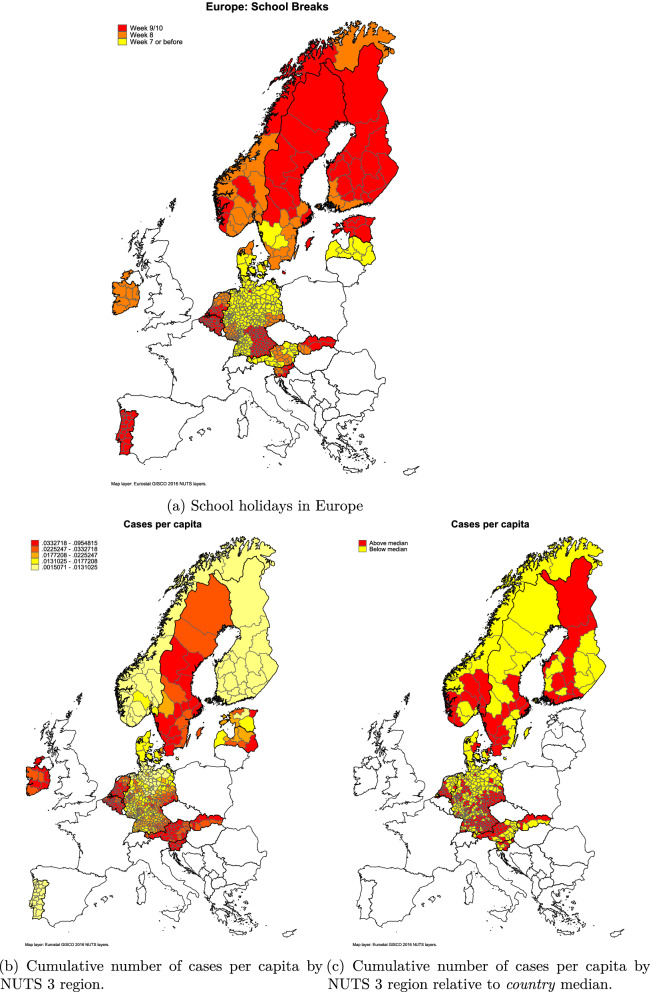
Comparison of school holidays and cumulative number of Covid-19 cases (until end of November 2020). Source: The data used to create figures (**b** and **c**) come from^9^. The source files for the map borders come from^10^. The maps are were created by the author using the statistical software package Stata, version 16 (see https://www.stata.com/).

From the maps in Fig. 3 we can see a comparison of the cumulative number of cases per capita up to end of November 2020 and a comparison with school holiday weeks. The map shows the number of cumulative cases of Covid-19 per capita (b), and the number of cases in a country relative to the median in that country (c). Investigating these maps, we can see some clear patterns. In the Netherlands, the southern part (week 9 holiday) has been harder hit than the northern week 8 holiday regions. Looking within Germany, regions with school holidays in weeks 8–10 have relatively higher number of cases of Covid-19. Belgium, the hardest-hit country in the EU, has a nationwide school holidays in week 9 as well as Stockholm, the badly affected capital of Sweden. See Tables D2, D3 and D4 in Appendix D for descriptive statistics on the school holiday weeks.

## Data and estimation

To investigate if school holidays in weeks 8, 9 and 10 led to large outbreaks in the spring and can explain subsequent spread of Covid-19, we first collect data on school holidays across Europe. The main source on school holidays is Eurydice network^11^established by the European Commission, which collects yearly information on the structure of the school year in Europe. This is then cross-referenced with other sources, see data Appendix A.

Several datasets on the spread of Covid-19 are used. First, we use data that has been collected and harmonized on case counts of Covid-19 at the NUTS 3 level for a number of European countries^9^. The NUTS classifications are a hierarchical system used in the EU based on administrative borders. NUTS 3 are small regions, NUTS 2 are larger areas, while NUTS 1 are major socio-economic regions. For the first part of the analysis, I use Covid-19 case numbers from 650 regions in 14 European countries. Secondly, data from RKI Germany is used for Covid-19 related deaths. See Appendix A for more information on data and Appendix B for more details on the selection of countries.

To assess if the timing of school holidays is important for the spread of Covid-19, we run a standard OLS regression to estimate Eq. (1). In this regression we try to explain the number of cases of Covid-19 per NUTS 3 region (ln number of cases) with a single joint dummy variable (*break*) for regions that have school holidays in weeks 8, 9 or 10.

1 $$\begin{aligned} ln(cases)_{r} = \beta _{1} break_{r} + region_{r} + CD_{c} +\epsilon _{r} \end{aligned}$$

I run a cross-sectional regression separately for each month (11 regressions) to investigate not only if late school holiday (weeks 8, 9 or 10) were important for the initial exposure, but also persistence over time. The cases are first aggregated to 7-day intervals and then to the monthly level, which roughly correspond to a month. A number of NUTS 3 specific control variables ($region_{r}$) from Eurostat are added. These include a categorical variable on urbanization (three categories predominantly urban, intermediate and predominantly rural), population, regional income, area(km sq.), median age and percentage of people below 14 and share above the age of 60. The inclusion of these variables controls for the cross-region demographics and importantly variation in population density in terms of number of inhabitants, typology (urban-rural) and geographic size. Errors are clustered at the NUTS 2 level.

Recall that in Eq. (1) we include dummy variables for regions that have a school holiday in either week 8, 9 or 10 (*break* equals 1 if region had a school holiday in either week 8, 9 or 10). In practice, this means that we are comparing regions that had a break in these higher exposure weeks to regions that had a school holiday break in week 7 or earlier (controlling for regional variables as noted above). In addition, we add a country specific dummy ($CD_c$) to the regression. As we know, testing strategies vary significantly *between* countries and the inclusion of a country specific effect accounts for such differences. By using a country specific dummy, we are effectively using variation *within* a country to identify the effects. Note that countries with the same school holiday profile may still experience a variation in the overall level of cases coming in to the country, stemming from the country specific propensity to travel abroad during the school holiday. See also a discussion in Appendix C (Table C1) on travel patterns to the known hot-spots in the alps in February and March 2020. As the response in the spring was mostly *country* specific, containment policy should not play a large role in the relative distribution of cases *within* a country. The country specific fixed effect will, for example, capture country specific lockdowns or other containment policies.

The identification strategy employed uses the fact that school holidays are decided long in advance and the exact timing is naturally exogenous to the spread of Covid-19 in February/March 2020. In addition, it is important to highlight two further aspects. First, by using country specific effects ($CD_{c}$) we exploit *only* regional within-country variation. Countries without variation in the timing of school holidays will therefore not contribute to the estimate of $\beta _{1}$ (Belgium for example only had a holiday in week 9). Second, regional characteristics ($region_{r}$) are added to account for observed regional differences (e.g. density, age profile). Hence, we compare regions which had a holiday in week 8 or later (“treated”) to regions, *within the same country*, that had a holiday in week 7 or before (“controls”) after accounting for observed country and regional level differences. Using Germany as an example, we compare regions in Bavaria (week 9) and Hamburg (week 10) to Berlin (week 6) and regions in North Rhine-Westphalia (no school holiday) after controlling for regional characteristics. To summarize, I argue that given the exogeneity of the exact timing of holidays, and controls for observed differences, we are able to infer the role of school holidays on the initial spread of Covid-19.

**Figure 4:**
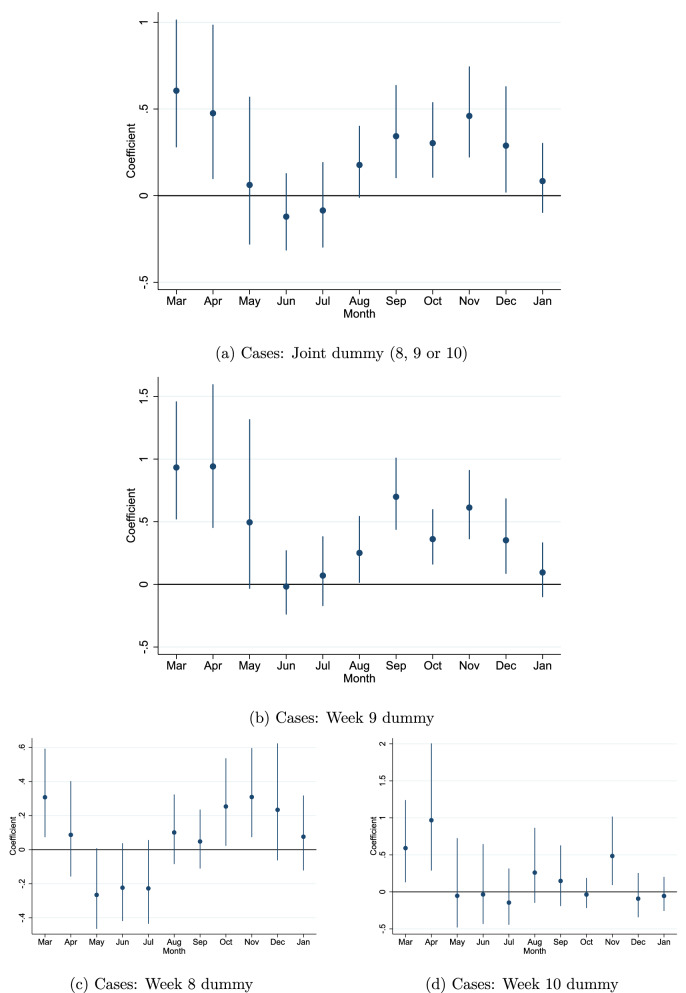
Coefficient plot of the joint dummy per month in graph (**a**) from March 2020 to January 2021. Sub-graphs (**c**), (**b**) and (**d**) show week 8, 9 and 10 dummies (in a single regression without the joint dummy). The coefficients in the diagrams have been transformed ($e(\beta _{1}-1$) for easier interpretation.

Figure 4 shows the OLS estimation results from these *eleven* monthly regressions. We can see clearly that regions with a school holiday in week 8, 9 or 10 had a considerably higher spread of Covid-19 in March-April. Quantitatively, the difference between late and early school holiday regions is large. We see that the number of cases in March is around 60% higher for regions with a school holiday in either week 8, 9 or 10 (compared to regions within the same country with a school holiday in week 7 or before).

A natural extension is to investigate if specific week numbers matter more than others, as we may expect from the timeline presented above. We can alter Eq. (1) and instead of a single post-week 7 dummy, include separate week specific dummies (*break*8 for week 8 etc.) as shown in Eq. (2).

2 $$\begin{aligned} ln(cases)_{r} = \beta _{1} break8_{r} + \beta _{2} break9_{r} +\beta _{3} break10_{r} + region_{r} + CD_{c} +\epsilon _{r} \end{aligned}$$

We estimate the equation with OLS as before using the same control variables. The coefficients for the week specific holiday break dummies are shown in Fig. 4b–d. We can see that the initial impact is strongest in regions with a school holiday break in week 9 (over 90%) secondly in week 10 (50–95%) and lowest in week 8 (35%). As before, these results are relative to regions that had a break in week 7 or before. Full results are available in Tables E5 and E6. It should be noted that, independently and parallel to this work, another study^12^ has used the variation in winter school holiday weeks to investigate policy effectiveness and the impact on excess deaths in the spring of 2020. Similarly, they find that week 9 school holiday led to a substantial share of the excess deaths in week 9 regions.

In some cases the school holiday vary *within* a NUTS 3 regions (e.g. Netherlands, Denmark and Sweden). Such variation will tend to attenuate the results presented above and bias the coefficients to zero, our results may therefore provide an underestimate/lower bound of the true effect in such cases.

One potential amplifying factor for regions with holidays in weeks 9 and 10 is that since Covid-19 had been introduced to a country by week 8 (9) travelers, international travel may not be needed, only travel to other infected regions within the country. Hence, *domestic travel* to newly infected regions with school holidays in earlier week(s) may have amplified the impact for regions with breaks in weeks 9 and 10. The spread will also be less clustered over time in Europe (e.g. away from ski-resorts) and travel to other destinations may become risky at this point. Examples of such events include an international business conference in Boston during week 9 (February 26–27)^13^. From analyzing genome sequences, some cases of Covid-19 were likely transmitted from Denmark to Sweden in March 2020^6^. This is consistent with school holidays potentially being important since the breaks in Denmark are mostly in weeks 7/8 while the receiving areas are mostly in northern Sweden (week 10). The results are also robust to adding gravity variables related to Ischgl and other related robustness checks. See Appendix C for a discussion.

### Persistence

Above we have established that school holidays in weeks 8, 9 and 10 are a strong indicator of large outbreaks of Covid-19 in the spring of 2020. The second main objective of this paper is to show how initial exposure is still relevant during the fall/winter of 2020. We can see from the Fig. 4a that despite the apparent disappearance of the initial outbreaks, the impact re-appears after the summer holidays. This can be seen from the re-emergence of a significant holiday week dummy from September and on-wards. On average, the spread is 30–50% higher in areas with high likelihood of initial exposure (holiday in week 8, 9 or 10).

Investigating the persistence separately by school holiday week, we can see an indication that the persistence is proportional to initial exposure, with the earliest and strongest resurgence in areas with school breaks in week 9 (40–70% higher). This suggests that the size of the outbreak impacts the degree of latent spread in an area, driving the systematic re-resurgence of Covid-19 in the fall of 2020. Large outbreaks will therefore have long-lasting consequences on community spread, since it may be difficult to capture or suppress the underlying (latent) spread fully.

The Global Initiative on Sharing All influenza Data (GISAID)^14^ provide a nomenclature system for genome sequences of SARS-CoV-2 that clusters strains/variants based on their genetic relatedness into eight main clades^15^. Investigating the broad clades in the GISAID database, we can see that the clade that dominated in a region in the spring 2020 tends to be the largest even after the summer, indicative of persistence. We can for example see that both in the spring and early fall the GH clade has dominated in Northern-America followed by, but to a much lesser extent, the G clade. This is in stark contrast with Europe were the GR clade dominates which accounts for a very low share of cases in Northern-America. Furthermore, in Europe, G and GH clades had a large share both in the spring and after the summer. In South America, the GR clade has dominated from the start. In late 2020, GISAID formed a new GK clade, consisting of the Delta variant (B.1.617.2). Note however that the sample period of this study ends in January 2021, which is well before the Delta variant (B.1.617.2) started to dominate in Europe.

One may be worried about regional containment measures impacting these results. However, it is important to note that such policies are generally skewed to areas with high levels of spread. As shown before, these are the areas that had holidays in weeks 8, 9 and 10. Regional containment policies would therefore tend to bias the results to zero, and the persistence would therefore be *stronger* if not for such policies. A number of hard hit countries/regions started to re-introduce stricter containment policies in September and October. While local authorities in Germany have autonomy in imposing restrictions, the German federal government put in place a trigger-based system were local authorities were advised to consider imposing lockdowns if new cases went above 50 per 100 thousand residents^16^. Germany introduced a nationwide “lockdown light” from the beginning of November and a full nationwide lockdown from mid-December. Note that two large regions, Bavaria (week 9), and Saxony (week 8) went into a stricter lockdown prior to the full lockdown which came into effect on December 16th. In the Netherlands, stricter measures were announced on September 18th for six security regions (of 25). All of which had (mainly) school holidays in week 9. On September 25th, eight more regions received the tighter measures, six of which had holidays in week 9, two in week 8. Soon thereafter, more national measures were introduced, a partial lockdown from mid-October and a full lockdown from mid-December. This provides indications that the persistence may be underestimated due to targeted regional policy in hard hit regions. A successful nationwide lockdown would also likely tend to reduce the overall level of the spread and hence observed regional difference, similarly to the convergence over the summer.

While the geographic footprint that the school holidays 2020 left behind is still visible in the fall/early winter, we would expect the systematic differences to subside as local outbreaks occur in new locations over time, attenuating the initial exposure. Hence, outbreaks in initially low exposure regions would also tend to lead to convergence over time and reduced importance of the school holidays. Investigating the results further, we can see how the impact of initial exposure decreases in December 2020 and January 2021 similar to the summer of 2020. This is consistent with that extensive containment measures had been in place for a substantial period in the largest countries in the sample (Germany and the Netherlands, see discussion above) and the introduction of other (potentially) more contagious strains which would naturally be unrelated to initial exposure.

Similarly, in a standard epidemiological SIR model, for example, infectious individuals pass on the virus to the susceptible population. Over time, as more and more people get infected, the population of susceptible individuals reduces, eventually slowing down the spread until herd-immunity is reached. In our setting, this would eventually lead to convergence between the initially hard hit areas and those less exposed, as herd immunity should be reached earlier in the highly exposed regions. However, as long as the share of people that are immune is fairly low, convergence between high- and low exposed areas would only occur gradually. To reach herd-immunity, a sizable share of the population needs to be immune to Covid-19, either by vaccination or antibodies. A recent study estimated that over 60% (and up to 90%) of the population would be needed to reach herd-immunity^17^. A Spanish nationwide study of over 51,000 individuals, conducted in November 2020, showed however that the share of people with antibodies was only 10% nationally and under 19% in the hardest-hit areas (see the ENE-COVID project^18^ and the fourth round results^19^). As Spain has experienced relatively large outbreaks of Covid-19 (see Fig. 1), it strongly suggests that the share of immune individuals is still well below the levels needed to reach herd immunity in both Spain and the rest of Europe during the period of interest.

### Covid-19 related deaths

It is well established that the number of Covid-19 tests varies between countries^2^. For our identification strategy, such cross-country variation in testing is captured by a country specific fixed effect ($CD_c$). Regional variation within a country may however impact the results if the number of tests is *disproportionately* higher in regions with a school holiday in weeks 8, 9 and 10. This could for example be due to better public access to testing in these regions. Using the school holiday weeks to identify regions with high likelihood of initial exposure is a strength of the study as we can overcome potential problems related to limited initial testing capacity in some countries (importantly not Germany) or in relatively more rural areas. Note also that if testing capacity was limited and, for example, regional capacity was reached during March/April this may tend to bias the results downward as the limit would be reached first in the high-exposure areas.

**Figure 5:**
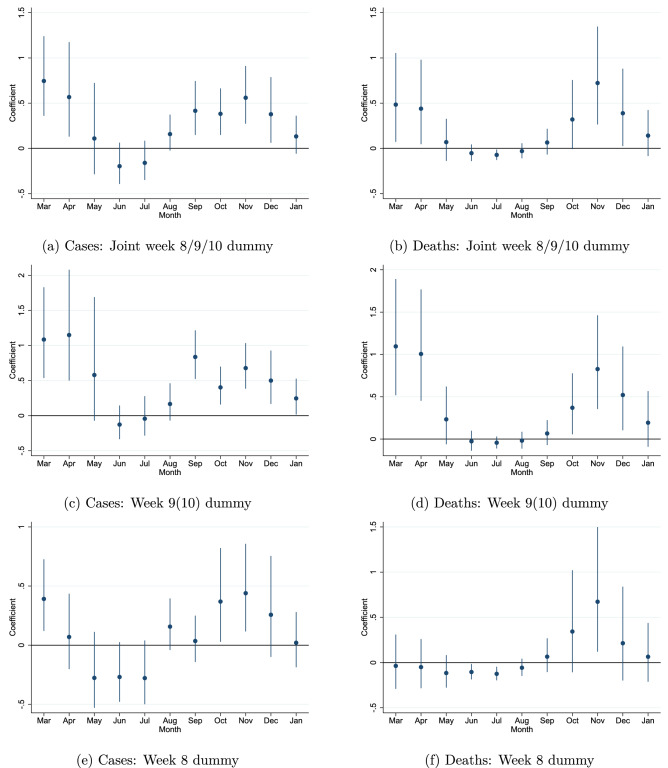
Germany only: results for the number of cases and deaths (joint week 8/9/10 dummy or separately week 8 and week 9(10) dummy. Robust standard errors clustered at NUTS 2 level.

This discussion underscores the importance of exploring the robustness of the results using other Covid-19 related outcomes. To investigate if (within-country) regional variation in testing impacts our results, we can use other outcomes which do not rely on public access, such as the number of Covid-19 related deaths. To investigate this, we use data from Germany. The German RKI publishes data on the number of cases and Covid-19 related deaths for each NUTS 3 region. Germany is particularly well suited for this purpose due to geographic and population size, large number of NUTS 3 areas, wide-spread testing from early stages and variation in timing of school holiday. Germany had homogeneous regional rules for testing^20^ and good relative initial testing capacity^21^. It is therefore natural to investigate it further. We can see from the sub-plots of Fig. 5, that the broad patterns are the same. Using the joint dummy model or separate week dummies (week 8 or week 9/10 combined) the results show the same trends. Full results are available in Tables E8, E9, E10 and E11 in the Appendix.

### Urban or rural persistence

**Figure 6:**
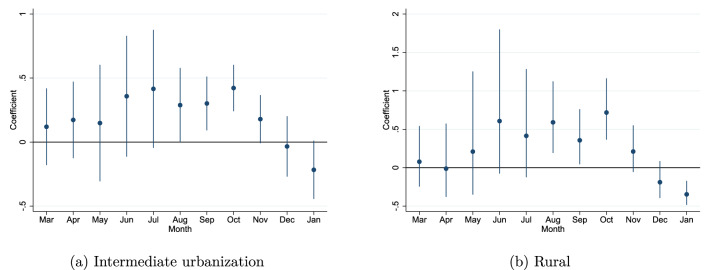
Urban/rural differences. Interaction of urbanization dummy and the joint week 8, 9 and 10 dummy. Results are transformed as before.

Urbanization is often discussed in relation to the spread of Covid-19 and many cities have experienced large outbreaks (e.g. New York, Madrid, Stockholm). A concern may therefore be that the result are driven by urban areas with high initial exposure. To investigate if and what role urbanization plays for the persistence of Covid-19 we add an interaction term for the joint holiday break dummy (week 8, 9 or 10) and our categorical variable for urbanization. The results show that the degree of persistence is *stronger* in *more* rural areas. It should be noted that the *level* of the spread of Covid-19 is higher in urban areas, but from the graphs we can see that the *persistence* related to initial exposure is higher (as can be seen from a significant interaction in the fall). The persistence is roughly 30% higher in intermediate urban areas and over 50% higher in rural areas in the fall. This suggests that Covid-19 persists even in smaller, more remote settings that experienced high initial exposure, and not only in urban settings. See Fig. 6 and Table E7. A potential explanation could be that containment policies have been more targeted at urban areas, and underestimated the potential persistence of Covid-19 in relatively rural communities.

## Conclusion

While the spread of Covid-19 was sporadic and localized in Europe at the beginning of February 2020 the coronavirus was spreading at an alarming rate by the end of the month. This paper explores the role of school holidays during this *key period* in sparking large initial outbreaks and persistence over time. The combination of the clustered nature (time/space) and *exact* timing of the school holidays during this period led to a vastly different likelihood of returning travelers transporting the virus back to their local community.

The two main contributions of the paper are the following. First, I find that having a winter school holidays from late-February (weeks 8, 9 and 10) led to 60% to over 90% higher *initial* spread of Covid-19 (compared to other regions in the same country). The results suggest that the school holidays in weeks 8, 9 and 10 were a region-wide super-spreading event. Secondly, even after the apparent containment of Covid-19 over the summer 2020 the same patterns re-emergence and the spread is consistently 30–40% and up to 70% higher in the regions with school holidays in weeks 8, 9 and 10. In particular, I find that regions with school holiday in week 9 experienced both the largest outbreaks in the spring the strongest resurgence from the fall (e.g. southern-Germany, southern-Netherlands, entire Belgium, Stockholm). The results therefore suggest that the underlying latent spread is relatively higher in areas that have previously experienced large outbreaks, even when such systematic differences are not visible (e.g. over the summer).

The main policy consequences of the results are that once a *large* outbreak of Covid-19 has occurred, it persists in the region. Rapid measures to avoid the *first* large outbreak are therefore fundamental to long-term containment. While avoiding large outbreaks of Covid-19 will entail short-term containment costs, the benefits may be long-lasting. The strong degree of regional persistence stresses the need for vigilance and effective vaccinations of the population to avoid further outbreaks. This applies especially to regions that have recently experienced large outbreaks but are well-below the levels needed for herd-immunity, as they may be particularly vulnerable. More broadly, the results are also indicative of the longer-term benefits of avoiding initial exposure to new, potentially more contagious, strains of the coronavirus.

Lastly, the winter school holidays provide an influential exogenous measure of initial exposure to Covid-19 for a number of European regions. This is particularly important to account for when evaluating the effectiveness of containment measures or other policies in the spring of 2020.

## Supplementary Information

Supplementary Information.

## Data Availability

The code and data files used for this paper are available^23^, see the data Appendix A for more information. This paper uses only publically available data and is conducted in line with the STROBE guidelines for cross-sectional studies. Note that a pre-print of this paper was called “Breaks and Breakouts: Explaining the Persistence of Covid-19”.
