# Supplementary material for: How a school holiday led to persistent COVID-19 outbreaks in Europe
Source: Sci Rep. 2021 Dec 22;11:24390. doi: 10.1038/s41598-021-03927-z (PMC8695576; doi:10.1038/s41598-021-03927-z)
Supplement: Supplementary file 1 — Supplementary Information. [file 41598_2021_3927_MOESM1_ESM.pdf]

Online Appendix to:

"How a school holiday led to initial COVID-19 outbreaks that persisted"

by

Björn Thor Arnarson

Department of Economics, University of Copenhagen

A Data Appendix

Naqvi [9] has collected and harmonized daily Covid-19 case data for a number of European countries at the NUTS 3 level. The 14 countries used for this analysis are the following: Austria (AT), Belgium (BE), Denmark (DK), Germany (DE), Estonia (EE), Finland (FI), Ireland (IE), Latvia (LV), Netherlands (NL), Norway (NO), Portugal (PT), Sweden (SE), Slovenia (SI) and Slovakia (SK). See table D2. The version 1.3. from 14th of February 2021 is used for this analysis. Documentation of the harmonization, change-log, code and original sources for each country can be found on the associated GitHub webpage. The German data comes from *The Robert Koch Institute* and includes the number of cases and deaths on a daily basis at the NUTS 3. Data on Germany is sourced from Gehrcke [24] on February 22nd, 2021. Alternative sources of school holiday break weeks are the websites www.Feiertagskalender.ch and Expatica. See Arnarson [23] for code and data files used for this paper.

B Country selection

This study limits the sample of countries used to those that plausibly had minimal exposure to Covid-19 prior to the school holidays in February and March 2020. The main criteria for inclusion are: 1) the regional variation in school holidays is clear and clustered in both time and space, and 2) the domestic spread did not take off due to other events during the holidays. In this section we discuss why some countries do not fulfill these criteria.

Large outbreaks during week 8 and 9 that can be traced to other events than school holidays would tend to create different spatial patterns and attenuate the observed impact of the winter school holidays. Examples of such events are discussed below. On February 19th the Champions

League football game between Atalanta and Valencia was played. This game, often dubbed as "Game Zero", is thought to have been a super-spreading event, sparking the initial spread in Northern Italy. The game was held two days before the first confirmed locally transmitted case in Italy. The number of confirmed cases in Bergamo, home of Atalanta, skyrocketed following the game and over a third of the Valencia team became infected [25]. In Spain the game is thought to contributed to the initial spread along with other events during the same week [26]. A gathering between 17-24th of February in Mulhouse is thought to have played a large role in the spread in France. Of the 2500 participants, at least half are thought to have become infected [27]. The closure of 120 schools in France was announced on March 3rd, located in three different regions spread over the country: Northwestern Morbihan, Oise north of Paris, and the department Haute-Savoie in eastern France [28].

In France, such early super-spreading events combined with that the schools holidays are two weeks long, and partly overlap, will tend to reduce the clustering (in time/space) and likely importance of winter school holidays for the spread. The domestic spread in Italy/Spain increased earlier, as is even visible from figure 2. The variation in school holidays is also unclear, while some regions/cities in both countries have some school holidays in part of week 9. Italy, Spain and France are therefore excluded from this analysis. A number of (eastern) European countries are also excluded that have unclear (or no) variation in school holidays (e.g. Romania, Croatia, Greece, Czech-Republic, Hungary, Luxembourg), lack of Covid-19 data at fine geographic level (Poland) or due to variation between municipalities in length and timing of holiday (e.g. in Iceland). UK is also excluded due to lack of school holiday variation and introduction of a new, more contagious, strain in the fall of 2020.

C Travel patterns, school holidays and distance

The state of Mecklenburg-Vorpommern in Germany has a school holiday that is somewhat unclear how to classify. The official dates for Mecklenburg-Vorpommern range from 10-21 February (defined as week 7) while, for comparison, the Saxony (official dates from 10-22 February) and Saarland (official dates 17-25 Feb) are both defined as week 8. There are several reasons why Mecklenburg-Vorpommern is classified as week 7. First, districts in Mecklenburg-Vorpommern are rather remote and furthest away from known initial hot-spots in the Alps. For our purposes,

since the holidays both ends first and the state is the most remote of the week 8 areas in Germany, this would likely lead to travelers *starting* their return earlier than those in other week 8 states in Germany (roughly 400 km south).

As discussed above in section 2 the spread was taking off during these pivotal days and finishing a school holiday earlier *within* week 8 may therefore lead to variation in likelihood of exposure. Secondly, the 2020 winter school holiday in Mecklenburg-Vorpommern started a week later than the three preceding years (begun on 4-6th of February). Saxony has for comparison begun breaks starting from 12-18th of February in previous years. If the timing of travel is somewhat sticky between years, it may lead to travel being relatively skewed to the early part of the holiday in Mecklenburg-Vorpommern while to the late half in Saxony/Saarland. To see if this classification impacts the results, we change the definition of Mecklenburg-Vorpommern to week 8 and the results are similar (impacts only week 8). The results are shown in appendix F, figure F1 and tables F12 and F13.

Austria is an example of a known hot-spot in late February and early March [29]. It is therefore natural to investigate the travel patterns to Austria during this period. From official Austrian tourism data [30] we can see that just below 2,7 million tourists visited the Austrian alps in February (three largest areas only: Tyrol, Salzburg, Vorarlberg). Investigating the origin country/region breakdown and duration of stay, we can see that the largest groups of visitors come from Germany, Netherlands, Belgium, Denmark and Sweden. Together they account for over 2 million visitors. From table C1 we can see considerable regional variation in the intensity of travel to Austria across German regions. Eastern-Germany and Berlin have the highest intensity of travel, as measured by average time spent in the Austrian Alps (120 and 116 number of nights per thousand inhabitants). A combination of many guests, relative to population, and long average duration is consistent with the fact that all the eastern regions and Berlin had extended holidays sometime during February. With the high intensity of travel but relatively early school holidays during (3-8 February, week 6), Berlin likely escaped a large initial outbreak.

We can also compare these regions to the similarly distant North Rhine-Westphalia and northern-Germany, which *do not* have a break in February. From table C1 we can see that the eastern regions and Berlin have a three-times higher level of travel (nights per capita), consistent with clustered school holiday travel. North Rhine-Westphalia and northern-Germany also share

Table C1: Number of visitors in Austria in February 2020 (March col. 7-8 only) and average duration by country/region of origin and relation to school-breaks.

	Nr. visitors	Nr. nights	Dura.	Per inhabitant Visitors	Per inhabitant Nights	March Dura.	March Nights	**Note on February school holiday**
Germany	1,339,005	6,237,466	4.7	16.1	75	5.8	28	-
Bavaria	*358,177*	*1,316,837*	*3.7*	*27.4*	*101*	3.7	33	Week 9
Baden Württemberg	*229,990*	*963,309*	*4.2*	*20.8*	*87*	4.2	26	None
North Rhine-Westphalia	*143,973*	*699,028*	*4.9*	*8.0*	*39*	7.8	24	None
Central Germany	*182,189*	*871,170*	*4.8*	*16.1*	*77*	5.8	31	Rheinland-Pfalz and Saarland (week 8)
Northern Germany	*104,397*	*494,772*	*4.7*	*7.8*	*37*	9.5	36	No extended breaks (two days at most)*
Eastern Germany	*243,683*	*1,454,867*	*6.0*	*19.4*	*116*	6.8	19	Breaks in all states during weeks 6-8
Berlin	*76,596*	*437,483*	*5.7*	*21.0*	*120*	6.9	22	Week 6
Belgium	104,636	596,238	5.7	9.0	51	5.9	9	Only week 9.
Denmark	86,885	480,160	5.5	15.0	83	8.6	13	Mostly week 7. Suburbs of CPH in week 8
Finland	9,743	47,737	4.9	1.8	9	6.9	3	Weeks 8-10. Helsinki week 8.
Netherlands	470,605	2,682,674	5.7	27.5	157	6.7	33	Week 8 or 9.
Norway	12,652	59,338	4.7	2.3	11	7	3	Weeks 8-10. Oslo week 8.
Sweden	42,323	240,827	5.7	4.2	24	8.4	5	Week 7-10. Stockholm week 9.

Note: The data from Statistics Austria groups German states in the following way: Central Germany: Hesse, Rhineland-Palatinate, Saarland. Northern Germany: Lower Saxony, Hamburg, Bremen, Schleswig-Holstein. Eastern Germany: Saxony, Saxony-Anhalt, Thuringia, Brandenburg, Mecklenburg-Vorpommern. The numbers in columns 5-6 and 8 are per inhabitant of the origin region/country (in thousands). Columns 7 and 8 (for March) can be compared to columns 4 and 6 (for February). *Hamburg in Northern Germany, is the only German NUTS 3 region that has a break in the beginning of *March.* See discussion in appendix C.

a border with the Netherlands and Denmark, which have much higher level of travel. From table C1 we can see that people from the Netherlands spend on average the most nights in the Austrian alps during February (157 nights per thousand inhabitants). A notable difference is that the Netherlands has a school holiday in week 8 or 9. We can even see high level of travel from Denmark during the winter school holiday season in February consistent, with Bluhm *et al.* [6] who trace the bulk of the genome sequences in Denmark to travel from Austria. Even in the south of Germany, relatively close to Austria, a difference can be seen in the travel between Bavaria (week 9) and Baden-Württemberg (none). While the numbers suggest some shorter day-travel to Austria, a clear difference in the number of guests and level of stay can still be seen from table C1 suggestive of clustered school holiday travel.

Note that Austria is just one of the most important destinations. Falk & Hagsten [4] find using contact tracing data from Norway, Sweden and Denmark that travelers from Italy and Austria accounted for the bulk of the initial inflow of cases. They find a large surge in cases between 12th and 24 of March, consistent with identifying cases following return home from winter-holiday traveling. The contact tracing data in Falk & Hagsten [4] is split by country pairs, which enables us to see the relative importance of the destinations. Austria accounts for most cases in Denmark (consistent with Bluhm *et al.* [6]) and Norway, while Italy is the largest origin country in Sweden, followed by Austria). Note that the country fixed effect will capture cross-country differences in preferences for destinations during the break.

Another way to analyze if travel is clustered during the school holiday is to zoom in on Hamburg in Northern-Germany. Regions in Northern-Germany do generally not have a school holiday in February, only Hamburg has a 2-week-long break in early March. Hence, if school

holidays influence travel patterns, we would expect this to be seen in the data for March. From table C1 (columns 7-8) we can see that in March 2020 the inhabitants of Northern-Germany had the longest duration of stay and spent the most nights per capita in March. The level is similar to February (36 v.s. 37) despite the general large drop in the number of tourist nights in Austria in March. This is a sharp difference from February, when Northern-Germany had the lowest level (see columns 6 and 8 in table C1). As Hamburg accounts for less than 15% of the total population of the Northern-region, this indicates that the travel from Hamburg was substantial. Hamburg has a school holiday in week 10 and 11 (defined as 10 in our analysis). As expected, the overall level of travel falls sharply in March compared to previous years, but we would expect the impact to be less pronounced in the very beginning of the month. We can investigate this hypothesis for Northern-Germany, as we expect people on school holiday in Hamburg to travel in that period. Investigating the data from, Statistics Austria [30] we can see that for March 2020, compared to March 2019, the number of nights spent in the Austrian alps decreased by only 24% for people coming from Northern-Germany. A reduction of 59% is seen for other regions of Germany. This is highly indicative of travel in the very beginning of March during the school holiday in Hamburg. As the travel from the region is expected to be clustered in the very beginning of the month, it should be less influenced by the events starting in week 11 (see section 2). This is not driven by variation in school holiday timing in Hamburg, as it also had a two weeks long school holiday in 2019 starting in early March. According to news reports, 80 cases in Hamburg had been traced to Ischgl in late March, and the resort was not fully quarantined until 13th of March [29].

Another pattern from table C1 is the very sharp decrease in the level of travel from Berlin and eastern-Germany in March. These two areas had the highest number of nights per inhabitant in February but the lowest in March, consistent with clustered travel during the school holiday weeks. Similarly, the level of travel is still relatively low for North Rhine-Westpalia which does not have a holiday in either February or March. Similarly, for other countries we can see the largest *relative* decline between February and March in the average number of nights per inhabitant in Denmark (mostly week 7 and partly 8) and smallest for Finland and Norway (weeks 8-10) consistent with the structure of the school holidays. This strongly suggests that the school-holidays have a substantial impact on when and for how long people travel to the

Austrian alps, regardless of distance.

Felbermayr *et al.* [31] estimate a gravity type model and find that distance alone to Ischgl in Austria explains a substantial fraction of the initial spread of Covid-19 in Germany (not using variation in school holiday timing). In our main specification a country fixed effect is included which will capture the absolute and country-specific part of the distance to Isghl. Adding distance to the regression would therefore only capture relative distance to Ischgl *within* a country. Above we have established that the travel patterns to Austria align well with travel during a school holiday and hence unclear what the relative distance would be capturing in the countries used in our analysis. However, to investigate if distance in general is important for the result, I perform a number of robustness checks.

First, one way to investigate the role of absolute and relative distance is to remove the country specific fixed effect. While not preferred for our main specification, it allows us to investigate the role of absolute distance on the results. Hence, we rerun the regression without the country specify fixed effect but both with, and without, distance to Ischgl. Distance between NUTS 3 areas is drawn from the European Commission's Tercet distance matrix, which has calculated the road distance between any two NUTS 3 areas. Distances are missing for two NUTS 3 regions in Portugal. The results show that distance does not impact the results once the country-fixed effect is removed, consistent with the timing of the school holiday driving the results rather than proximity. See figure F2 and tables F14 and F15.

Second, as noted above, by including distance to Ischgl we can also investigate the role of relative within country distance to Ischgl. After adding distance to Ischgl and including the country specific fixed effect, the results are broadly similar. Note that initial impact in March/April is still large and highly significant for week 9 (around 50%) but appears somewhat dampened. The large week 10 effect is unchanged. This likely reflects the widespread testing in Germany from the first initial stages, compared to other countries, combined with the fact that week 9 regions in Germany are in the most southern part of the country (relatively closest to Ischgl). Importantly, we see that the results on post-summer persistence are unchanged, consistent with the results in figure F2 which shows minimal role of absolute distance. See figure F3 and tables F14 and F15 for the full results. The inclusion of distance creates some odd patterns when used with multiple countries, as regions that have the same absolute distance to

Ischgl will be considered relatively far and relatively close, depending on the domestic internal distance. This is since the country fixed effect will capture the part of the distance that is common to all regions in a country. An example is Netherlands and Denmark, who border Germany. Many of these areas will have the lowest internal distance, while the bordering areas in Germany will have relatively high measure of internal distance, since other German areas are closer.

Third, we may be worried that the results are driven by German regions that are within a few hundred kilometers from the Austrian ski-resorts. Hence, regions where it may be possible to take day-trips to the Alps rather than longer extended periods during the school holidays. To investigate this potential issue, we drop the two regions in Germany that are closest to Austria (Bavaria and Baden-Württemberg). The results are very similar, see figure F4. Fourth, as Covid-19 spread over time to more areas, travel to non-ski related areas may lead to exposure in the later holiday break weeks (9-10).

Distance is therefore not included in the main specification since: 1) the absolute distance has a no impact on the results suggestive of a dominant role of the timing of travel, 2) the relative internal distance to Ischgl has in general a small and unclear role in our setting given country fixed effects and other controls (e.g. typology), 3) the spread becomes more broad over time resulting in travel in general being likely to lead to exposure and not only travel to Austria.

D Descriptive Statistics

Table D2: Share of regions in each holiday week by country (upper panel) and share of population in each break-week (lower panel).

	Country code													
	AT	BE	DE	DK	EE	FI	IE	LV	NL	NO	PT	SE	SI	SK
<=W7	0.69	0	0.62	0.73	0	0	0	1	0	0	0	0.095	0	0
W8	0.31	0	0.14	0.18	0	0.21	1	0	0.50	0.72	0	0.43	0.33	0.38
W9	0	1	0.24	0.091	1	0.53	0	0	0.50	0.17	1	0.29	0.67	0.38
W10	0	0	0.0025	0	0	0.26	0	0	0	0.11	0	0.19	0	0.25
	Country code													
	AT	BE	DE	DK	EE	FI	IE	LV	NL	NO	PT	SE	SI	SK
<=W7	0.69	0	0.71	0.85	0	0	0	1	0	0	0	0.2	0	0
W8	0.31	0	0.11	0.14	0	0.43	1	0	0.36	0.67	0	0.36	0.47	0.35
W9	0	1	0.16	0.01	1	0.37	0	0	0.64	0.24	1	0.35	0.53	0.35
W10	0	0	0.02	0	0	0.19	0	0	0	0.08	0	0.09	0	0.30
# NUTS 3	35	44	401	11	5	19	8	6	40	18	25	21	12	8

Table D3: Descriptive statistics for all countries

	Week nr.			
	<=7	8	9	10
Population	256054	277042	258720	417512
Median age	46.3	44.6	44.5	43.0
Area (km sq.)	1478	3346	2277	34639
Income	9602	1087	9174	15770
Share over age 60	28.4	27.3	27.0	27.3

Table D4: Degree of area urbanization by school holiday week (column %)

	Week Nr.				
Deg. of Urbanization	<=7	8	9	10	Total
Cities/Urban	26	22	19	7	23
Predom. Urban	48	46	39	29	44
Rural	26	32	41	64	33

E Results Appendix

E.1 Broad Sample Results

Table E5: Baseline results when using a joint dummy (either week 8, 9 or 10).

	Mar	Apr	May	Jun	Jul	Aug	Sep	Oct	Nov	Dec	Jan
Break =>W8	0.473^a	0.389^b	0.0601	-0.129	-0.0892	0.163^c	0.295^a	0.265^a	0.378^a	0.253^b	0.0807
	(0.115)	(0.150)	(0.198)	(0.126)	(0.134)	(0.0883)	(0.100)	(0.0838)	(0.0901)	(0.119)	(0.0933)
Population	0.500^a	0.947^a	0.923^a	0.787^a	0.981^a	0.774^a	1.110^a	1.262^a	1.252^a	1.228^a	1.205^a
	(0.127)	(0.178)	(0.202)	(0.227)	(0.213)	(0.130)	(0.114)	(0.0982)	(0.106)	(0.119)	(0.100)
Median age	5.663^a	9.961^a	9.832^a	6.540^a	5.340^b	5.627^a	2.084	0.113	2.391^b	2.301^c	0.359
	(1.387)	(1.535)	(1.687)	(2.431)	(2.233)	(1.413)	(1.537)	(1.269)	(1.192)	(1.331)	(1.050)
Share below 14	-0.142	-0.0580	0.379	1.197^c	1.351^b	0.701	1.344^a	1.071^a	0.448	-0.152	-0.0102
	(0.536)	(0.660)	(0.878)	(0.653)	(0.650)	(0.497)	(0.504)	(0.388)	(0.430)	(0.446)	(0.338)
Share over age 60	-4.565^a	-6.340^a	-5.488^a	-3.281^b	-3.484^b	-4.824^a	-2.525^a	-1.037	-1.428^c	-0.445	0.997
	(0.839)	(0.931)	(1.105)	(1.504)	(1.500)	(0.889)	(0.894)	(0.758)	(0.848)	(1.021)	(0.726)
Area (km sq.)	0.0189	-0.183^a	-0.245^a	-0.293^a	-0.248^a	-0.199^a	-0.254^a	-0.0980^a	-0.120^a	-0.122^a	-0.0698^b
	(0.0424)	(0.0503)	(0.0609)	(0.0738)	(0.0662)	(0.0432)	(0.0393)	(0.0351)	(0.0309)	(0.0319)	(0.0348)
Income	0.557^a	0.321^a	0.311^b	0.415^b	0.277^c	0.403^a	0.168^c	-0.100	-0.0981	-0.0551	-0.0547
	(0.0999)	(0.121)	(0.153)	(0.175)	(0.156)	(0.0951)	(0.0990)	(0.0859)	(0.0806)	(0.0883)	(0.0701)
Interm. urb.	0.0158	0.0932	-0.0537	-0.166	-0.237^c	-0.172^b	-0.0274	-0.194^a	-0.133^b	-0.0166	0.0284
	(0.0879)	(0.0995)	(0.135)	(0.145)	(0.140)	(0.0802)	(0.0677)	(0.0698)	(0.0567)	(0.0606)	(0.0553)
Rural	-0.00222	0.318^c	0.0537	-0.0967	-0.137	-0.212^c	0.0192	-0.138	-0.0736	0.0415	0.132^c
	(0.109)	(0.162)	(0.214)	(0.194)	(0.170)	(0.121)	(0.107)	(0.0981)	(0.0900)	(0.0876)	(0.0789)
BE	0.251	1.972^a	2.454^a	0.857^b	0.447	0.214	-0.194	0.687^a	-1.220^a	-0.766^a	-0.404^b
	(0.256)	(0.281)	(0.378)	(0.333)	(0.271)	(0.173)	(0.222)	(0.207)	(0.180)	(0.184)	(0.155)
DE	0.0166	0.886^a	1.134^a	-0.0414	-0.636^a	-0.181^c	-1.100^a	-1.030^a	-1.390^a	-0.388^a	-0.323^a
	(0.204)	(0.221)	(0.285)	(0.198)	(0.166)	(0.101)	(0.168)	(0.0991)	(0.125)	(0.124)	(0.119)
DK	-0.250	1.141^a	1.688^a	0.446	-0.362	-0.137	-0.0445	-1.146^a	-1.376^a	0.303^c	-0.458^a
	(0.262)	(0.324)	(0.411)	(0.400)	(0.316)	(0.353)	(0.206)	(0.133)	(0.178)	(0.182)	(0.141)
EE	-1.021^a	0.585^c	1.595^a	0.376	-1.005^a	-0.413^b	-0.451^c	-2.369^a	-1.651^a	0.0524	0.506^a
	(0.267)	(0.299)	(0.367)	(0.372)	(0.353)	(0.208)	(0.269)	(0.182)	(0.182)	(0.217)	(0.169)
FI	-1.242^a	0.409	1.428^a	-0.311	-1.238^a	-0.925^a	-1.304^a	-2.481^a	-3.002^a	-2.197^a	-2.011^a
	(0.298)	(0.329)	(0.448)	(0.432)	(0.398)	(0.213)	(0.308)	(0.191)	(0.245)	(0.315)	(0.231)
IE	-1.063^a	1.971^a	2.599^a	0.202	-1.050^a	-0.653^a	-1.103^a	-1.412^a	-2.817^a	-0.635^b	1.339^a
	(0.313)	(0.393)	(0.578)	(0.412)	(0.326)	(0.249)	(0.317)	(0.259)	(0.232)	(0.248)	(0.203)
LV	-1.279^a	0.0813	1.470^a	0.113	-1.076^a	-0.639^a	-1.900^a	-1.406^a	-1.265^a	0.351^c	0.731^a
	(0.232)	(0.265)	(0.372)	(0.325)	(0.306)	(0.188)	(0.235)	(0.163)	(0.176)	(0.187)	(0.162)
NL	-1.009^a	1.546^a	1.421^a	0.201	-0.909^a	-0.120	-0.289	-0.206	-1.415^a	0.233	0.322^b
	(0.282)	(0.299)	(0.403)	(0.351)	(0.315)	(0.193)	(0.217)	(0.149)	(0.168)	(0.183)	(0.146)
NO	-1.669^a	0.0683	0.809^c	-0.0899	-1.492^a	-0.775^a	-1.611^a	-2.373^a	-2.462^a	-1.689^a	-1.224^a
	(0.304)	(0.316)	(0.479)	(0.369)	(0.365)	(0.268)	(0.262)	(0.207)	(0.237)	(0.356)	(0.294)
PT	-1.068^b	1.183^a	2.342^a	1.909^a	-0.0425				-3.175^a	-1.965^a	-0.832^a
	(0.477)	(0.395)	(0.388)	(0.462)	(0.393)				(0.293)	(0.246)	(0.145)
SE	-0.612^c	2.558^a	4.698^a	4.262^a	1.668^a	1.028^a	-0.336	-1.005^a	-0.449^c	0.768^a	0.480^b
	(0.346)	(0.392)	(0.450)	(0.460)	(0.448)	(0.319)	(0.299)	(0.212)	(0.238)	(0.264)	(0.212)
SI	-0.804^b	-0.413	-0.234	1.145^a	0.448	0.311	-0.0468	0.314^b	-0.255	0.771^a	1.118^a
	(0.316)	(0.365)	(0.336)	(0.307)	(0.280)	(0.206)	(0.281)	(0.136)	(0.180)	(0.215)	(0.136)
SK	-2.424^a	-0.604^c	-0.404	0.0377	-0.474	-0.338	-0.444	-0.436^b	-1.336^a	0.344^c	0.660^a
	(0.280)	(0.325)	(0.421)	(0.454)	(0.301)	(0.285)	(0.293)	(0.191)	(0.199)	(0.192)	(0.179)
Constant	-12.10^a	-26.58^a	-30.71^a	-25.57^a	-21.24^a	-14.70^a	-11.08^a	-6.223^b	-10.92^a	-13.09^a	-11.13^a
	(3.331)	(4.037)	(4.824)	(4.626)	(4.633)	(2.881)	(3.482)	(2.824)	(2.563)	(2.191)	(2.171)
Observations	648	650	650	650	650	625	625	625	650	650	650

Standard errors, clustered at NUTS 2 level, in parenthesis. ^{c} $p < .1$, ^{b} $p < .05$, ^{a} $p < .01$
Same results as in figure 4, 4a. Data from two areas is missing in March and from Portugal in August-October.

Table E6: Baseline results using separate week specific dummies(w8, w9 and w10)

	Mar	Apr	May	Jun	Jul	Aug	Sep	Oct	Nov	Dec	Jan
Break W8	0.268^a	0.0832	-0.309^c	-0.253^c	-0.258	0.0961	0.0470	0.226^b	0.269^a	0.210	0.0732
	(0.0996)	(0.129)	(0.160)	(0.146)	(0.158)	(0.0931)	(0.0828)	(0.103)	(0.100)	(0.139)	(0.102)
Break W9	0.659^a	0.663^a	0.402^c	-0.0171	0.0679	0.224^b	0.530^a	0.308^a	0.478^a	0.302^a	0.0910
	(0.122)	(0.147)	(0.221)	(0.130)	(0.130)	(0.107)	(0.0850)	(0.0813)	(0.0859)	(0.111)	(0.0998)
Break W10	0.464^a	0.677^a	-0.0549	-0.0357	-0.157	0.231	0.137	-0.0373	0.395^b	-0.0963	-0.0572
	(0.173)	(0.214)	(0.303)	(0.269)	(0.218)	(0.198)	(0.176)	(0.105)	(0.155)	(0.163)	(0.122)
Population	0.569^a	1.108^a	1.097^a	0.851^a	1.060^a	0.810^a	1.223^a	1.265^a	1.305^a	1.233^a	1.203^a
	(0.135)	(0.176)	(0.191)	(0.234)	(0.212)	(0.130)	(0.110)	(0.100)	(0.106)	(0.112)	(0.0953)
Median age	4.982^a	9.109^a	8.461^a	6.177^b	4.701^b	5.428^a	1.000	-0.292	2.024	1.891	0.231
	(1.414)	(1.588)	(1.736)	(2.460)	(2.219)	(1.440)	(1.546)	(1.317)	(1.224)	(1.341)	(1.068)
Share below 14	0.0932	0.297	0.784	1.340^b	1.535^b	0.787	1.640^a	1.105^a	0.570	-0.122	-0.00892
	(0.510)	(0.595)	(0.803)	(0.641)	(0.627)	(0.488)	(0.471)	(0.391)	(0.429)	(0.444)	(0.337)
Share over age 60	-3.935^a	-5.472^a	-4.311^a	-2.922^c	-2.941^b	-4.619^a	-1.623^c	-0.798	-1.095	-0.212	1.060
	(0.869)	(0.965)	(1.119)	(1.504)	(1.462)	(0.882)	(0.901)	(0.791)	(0.879)	(1.026)	(0.752)
Area (km sq.)	0.0122	-0.214^a	-0.261^a	-0.305^a	-0.255^a	-0.206^a	-0.261^a	-0.0852^b	-0.127^a	-0.108^a	-0.0639^c
	(0.0428)	(0.0461)	(0.0599)	(0.0765)	(0.0690)	(0.0432)	(0.0369)	(0.0374)	(0.0309)	(0.0319)	(0.0353)
Income	0.503^a	0.202^c	0.174	0.367^b	0.214	0.376^a	0.0789	-0.108	-0.139^c	-0.0657	-0.0552
	(0.106)	(0.120)	(0.142)	(0.179)	(0.157)	(0.0970)	(0.0952)	(0.0856)	(0.0808)	(0.0840)	(0.0675)
Interm. urb.	0.00202	0.0920	-0.0764	-0.168	-0.248^c	-0.172^b	-0.0477	-0.211^a	-0.137^b	-0.0347	0.0217
	(0.0841)	(0.0950)	(0.127)	(0.145)	(0.134)	(0.0800)	(0.0663)	(0.0699)	(0.0556)	(0.0599)	(0.0559)
Rural	-0.0397	0.290^c	-0.00421	-0.110	-0.164	-0.217^c	-0.0222	-0.159	-0.0881	0.0178	0.124
	(0.100)	(0.158)	(0.208)	(0.197)	(0.162)	(0.120)	(0.104)	(0.0989)	(0.0876)	(0.0874)	(0.0800)
BE	-0.0894	1.445^a	1.821^a	0.644^c	0.157	0.0959	-0.628^a	0.621^a	-1.407^a	-0.838^a	-0.416^b
	(0.245)	(0.258)	(0.395)	(0.346)	(0.280)	(0.196)	(0.200)	(0.211)	(0.181)	(0.182)	(0.169)
DE	-0.104	0.685^a	0.913^a	-0.122	-0.736^a	-0.226^b	-1.246^a	-1.039^a	-1.457^a	-0.397^a	-0.321^a
	(0.185)	(0.192)	(0.245)	(0.203)	(0.188)	(0.102)	(0.139)	(0.103)	(0.127)	(0.128)	(0.122)
DK	-0.370	0.960^a	1.469^a	0.373	-0.462	-0.179	-0.203	-1.173^a	-1.440^a	0.276	-0.463^a
	(0.234)	(0.287)	(0.374)	(0.404)	(0.334)	(0.357)	(0.190)	(0.136)	(0.173)	(0.184)	(0.143)
EE	-1.400^a	0.0129	0.863^b	0.142	-1.342^a	-0.541^b	-0.962^a	-2.475^a	-1.863^a	-0.0639	0.479^a
	(0.267)	(0.289)	(0.376)	(0.383)	(0.353)	(0.228)	(0.251)	(0.195)	(0.188)	(0.210)	(0.182)
FI	-1.469^a	0.0322	1.043^b	-0.461	-1.412^a	-1.012^a	-1.569^a	-2.485^a	-3.123^a	-2.192^a	-1.999^a
	(0.290)	(0.316)	(0.476)	(0.437)	(0.384)	(0.209)	(0.305)	(0.183)	(0.253)	(0.294)	(0.235)
IE	-0.958^a	2.130^a	2.773^a	0.265	-0.971^a	-0.620^b	-0.994^a	-1.405^a	-2.764^a	-0.627^b	1.338^a
	(0.287)	(0.353)	(0.520)	(0.411)	(0.346)	(0.247)	(0.272)	(0.251)	(0.227)	(0.250)	(0.208)
LV	-1.475^a	-0.236	1.069^a	-0.0171	-1.261^a	-0.709^a	-2.179^a	-1.460^a	-1.381^a	0.290	0.717^a
	(0.217)	(0.236)	(0.325)	(0.331)	(0.309)	(0.189)	(0.213)	(0.171)	(0.179)	(0.185)	(0.162)
NL	-1.133^a	1.311^a	1.149^a	0.106	-1.033^a	-0.174	-0.478^b	-0.228	-1.497^a	0.210	0.320^b
	(0.243)	(0.251)	(0.353)	(0.337)	(0.300)	(0.192)	(0.196)	(0.142)	(0.160)	(0.177)	(0.147)
NO	-1.657^a	0.101	0.852^c	-0.0766	-1.471^a	-0.769^a	-1.590^a	-2.370^a	-2.449^a	-1.681^a	-1.222^a
	(0.287)	(0.288)	(0.452)	(0.374)	(0.369)	(0.263)	(0.229)	(0.205)	(0.247)	(0.352)	(0.292)
PT	-1.396^a	0.670^c	1.707^a	1.700^a	-0.334				-3.361^a	-2.051^a	-0.849^a
	(0.483)	(0.390)	(0.388)	(0.473)	(0.407)				(0.302)	(0.246)	(0.159)
SE	-0.762^b	2.322^a	4.445^a	4.167^a	1.553^a	0.972^a	-0.518^c	-1.018^a	-0.527^b	0.762^a	0.485^b
	(0.339)	(0.373)	(0.423)	(0.450)	(0.432)	(0.314)	(0.270)	(0.213)	(0.242)	(0.267)	(0.217)
SI	-0.998^a	-0.730^a	-0.618^c	1.016^a	0.272	0.241	-0.311^c	0.271^b	-0.369^b	0.725^a	1.109^a
	(0.234)	(0.239)	(0.320)	(0.350)	(0.250)	(0.188)	(0.182)	(0.132)	(0.148)	(0.194)	(0.140)
SK	-2.541^a	-0.887^a	-0.645	-0.0720	-0.581^c	-0.402	-0.589^b	-0.397^b	-1.418^a	0.390^b	0.685^a
	(0.280)	(0.328)	(0.442)	(0.445)	(0.319)	(0.310)	(0.289)	(0.177)	(0.207)	(0.188)	(0.178)
Constant	-12.39^a	-27.65^a	-31.01^a	-25.96^a	-21.34^a	-14.97^a	-11.11^a	-5.578^c	-11.11^a	-12.40^a	-10.85^a
	(3.184)	(3.772)	(4.585)	(4.758)	(4.783)	(2.939)	(3.450)	(2.892)	(2.538)	(2.165)	(2.152)
Observations	648	650	650	650	650	625	625	625	650	650	650

Standard errors, clustered at NUTS 2 level, in parenthesis. ^{c} $p < .1$, ^{b} $p < .05$, ^{a} $p < .01$
Same results as in figure 4, 4c, 4b and 4d. Data from two areas is missing in March and from Portugal in August-October.

Table E7: Urban/Rural comparison results

	Mar	Apr	May	Jun	Jul	Aug	Sep	Oct	Nov	Dec	Jan
Break =>W8	0.395^a	0.312^b	-0.0680	-0.443^b	-0.391^c	-0.116	0.0589	-0.101	0.236^b	0.334^a	0.314^a
	(0.143)	(0.133)	(0.195)	(0.223)	(0.226)	(0.121)	(0.111)	(0.109)	(0.0951)	(0.0936)	(0.0769)
Interm. urb.	-0.0443	0.00393	-0.128	-0.345^c	-0.447^b	-0.317^a	-0.179^b	-0.405^a	-0.223^a	-0.00163	0.135^c
	(0.117)	(0.131)	(0.185)	(0.206)	(0.203)	(0.0963)	(0.0841)	(0.0734)	(0.0683)	(0.0820)	(0.0784)
Rural	-0.0385	0.335	-0.0506	-0.358	-0.319	-0.470^a	-0.144	-0.433^a	-0.177	0.164	0.372^a
	(0.144)	(0.251)	(0.334)	(0.257)	(0.230)	(0.139)	(0.133)	(0.125)	(0.120)	(0.119)	(0.0920)
Break =>W8 × Interm. urb	0.120	0.173	0.148	0.358	0.415^c	0.289^c	0.302^a	0.422^a	0.179^c	-0.0341	-0.217^c
	(0.151)	(0.151)	(0.229)	(0.238)	(0.233)	(0.146)	(0.106)	(0.0913)	(0.0947)	(0.119)	(0.115)
Break =>W8 × Rural	0.0747	-0.0126	0.190	0.474^c	0.347	0.464^a	0.305^b	0.541^a	0.191	-0.210	-0.426^a
	(0.181)	(0.235)	(0.314)	(0.280)	(0.242)	(0.146)	(0.132)	(0.116)	(0.125)	(0.148)	(0.120)
Population	0.498^a	0.941^a	0.909^a	0.752^a	0.951^a	0.733^a	1.080^a	1.213^a	1.237^a	1.238^a	1.232^a
	(0.126)	(0.179)	(0.209)	(0.222)	(0.214)	(0.125)	(0.114)	(0.0892)	(0.105)	(0.117)	(0.0942)
Median age	5.587^a	9.807^a	9.791^a	6.450^a	5.143^b	5.440^a	1.892	-0.158	2.325^c	2.235^c	0.351
	(1.385)	(1.533)	(1.697)	(2.338)	(2.155)	(1.329)	(1.498)	(1.231)	(1.202)	(1.318)	(0.981)
Share below 14	-0.116	-0.0139	0.410	1.270^c	1.443^b	0.743	1.401^a	1.143^a	0.486	-0.153	-0.0489
	(0.546)	(0.668)	(0.881)	(0.661)	(0.659)	(0.502)	(0.504)	(0.406)	(0.436)	(0.453)	(0.327)
Share over age 60	-4.516^a	-6.265^a	-5.437^a	-3.157^b	-3.328^b	-4.640^a	-2.377^a	-0.801	-1.363	-0.446	0.931
	(0.850)	(0.928)	(1.102)	(1.453)	(1.456)	(0.841)	(0.863)	(0.744)	(0.860)	(1.001)	(0.677)
Area (km sq.)	0.0216	-0.178^a	-0.240^a	-0.282^a	-0.235^a	-0.185^a	-0.243^a	-0.0804^b	-0.114^a	-0.123^a	-0.0775^b
	(0.0424)	(0.0507)	(0.0647)	(0.0722)	(0.0650)	(0.0401)	(0.0394)	(0.0339)	(0.0306)	(0.0305)	(0.0320)
Income	0.559^a	0.323^a	0.322^b	0.442^b	0.298^c	0.432^a	0.188^c	-0.0651	-0.0868	-0.0648	-0.0767
	(0.0998)	(0.122)	(0.157)	(0.172)	(0.158)	(0.0942)	(0.0978)	(0.0785)	(0.0808)	(0.0893)	(0.0690)
BE	0.256	1.987^a	2.458^a	0.868^a	0.467^c	0.223	-0.179	0.705^a	-1.212^a	-0.760^a	-0.405^a
	(0.254)	(0.272)	(0.383)	(0.327)	(0.266)	(0.169)	(0.220)	(0.200)	(0.180)	(0.177)	(0.143)
DE	0.0246	0.917^a	1.130^a	-0.0520	-0.618^a	-0.199^c	-1.093^a	-1.037^a	-1.388^a	-0.362^a	-0.296^a
	(0.205)	(0.216)	(0.302)	(0.204)	(0.165)	(0.109)	(0.177)	(0.110)	(0.127)	(0.118)	(0.111)
DK	-0.253	1.155^a	1.671^a	0.401	-0.384	-0.186	-0.0682	-1.197^a	-1.391^a	0.331^c	-0.410^a
	(0.261)	(0.313)	(0.418)	(0.392)	(0.322)	(0.374)	(0.213)	(0.154)	(0.180)	(0.180)	(0.135)
EE	-1.019^a	0.610^b	1.586^a	0.353	-1.001^a	-0.450^b	-0.458^c	-2.399^a	-1.656^a	0.0797	0.542^a
	(0.270)	(0.290)	(0.380)	(0.371)	(0.340)	(0.200)	(0.264)	(0.182)	(0.187)	(0.207)	(0.154)
FI	-1.255^a	0.417	1.390^a	-0.407	-1.304^a	-1.037^a	-1.370^a	-2.606^a	-3.039^a	-2.151^a	-1.922^a
	(0.308)	(0.334)	(0.473)	(0.442)	(0.382)	(0.202)	(0.305)	(0.201)	(0.256)	(0.302)	(0.208)
IE	-1.064^a	1.990^a	2.583^a	0.160	-1.066^a	-0.691^a	-1.118^a	-1.449^a	-2.831^a	-0.604^b	1.387^a
	(0.313)	(0.392)	(0.589)	(0.406)	(0.323)	(0.252)	(0.304)	(0.237)	(0.230)	(0.255)	(0.187)
LV	-1.277^a	0.0920	1.476^a	0.127	-1.056^a	-0.629^a	-1.887^a	-1.389^a	-1.257^a	0.352^c	0.724^a
	(0.231)	(0.258)	(0.377)	(0.321)	(0.302)	(0.186)	(0.235)	(0.166)	(0.178)	(0.185)	(0.157)
NL	-0.993^a	1.567^a	1.447^a	0.265	-0.843^b	-0.0583	-0.234	-0.123	-1.385^a	0.220	0.277^b
	(0.280)	(0.292)	(0.407)	(0.354)	(0.328)	(0.201)	(0.223)	(0.160)	(0.170)	(0.173)	(0.134)
NO	-1.680^a	0.0671	0.780	-0.162	-1.547^a	-0.852^a	-1.662^a	-2.464^a	-2.491^a	-1.660^a	-1.162^a
	(0.308)	(0.316)	(0.500)	(0.385)	(0.346)	(0.248)	(0.260)	(0.209)	(0.249)	(0.347)	(0.264)
PT	-1.062^b	1.221^a	2.333^a	1.885^a	-0.0293				-3.178^a	-1.929^a	-0.788^a
	(0.481)	(0.390)	(0.401)	(0.454)	(0.386)				(0.299)	(0.240)	(0.142)
SE	-0.634^c	2.534^a	4.661^a	4.173^a	1.581^a	0.936^a	-0.409	-1.123^a	-0.490^b	0.789^a	0.545^a
	(0.351)	(0.390)	(0.468)	(0.457)	(0.433)	(0.304)	(0.296)	(0.214)	(0.247)	(0.264)	(0.201)
SI	-0.804^b	-0.382	-0.254	1.093^a	0.435	0.251	-0.0680	0.257^c	-0.270	0.815^a	1.182^a
	(0.324)	(0.376)	(0.355)	(0.317)	(0.282)	(0.197)	(0.286)	(0.139)	(0.186)	(0.222)	(0.129)
SK	-2.426^a	-0.594^c	-0.404	0.0368	-0.467	-0.336	-0.437	-0.429^b	-1.334^a	0.352^c	0.667^a
	(0.283)	(0.320)	(0.425)	(0.453)	(0.302)	(0.294)	(0.297)	(0.181)	(0.196)	(0.184)	(0.164)
Constant	-12.00^a	-26.32^a	-30.70^a	-25.55^a	-21.01^a	-14.41^a	-10.77^a	-5.791^b	-10.86^a	-12.92^a	-11.00^a
	(3.324)	(4.043)	(4.911)	(4.590)	(4.701)	(2.741)	(3.453)	(2.741)	(2.523)	(2.204)	(2.069)
Observations	648	650	650	650	650	625	625	625	650	650	650

Standard errors in parenthesis. ^{c} $p < .1$, ^{b} $p < .05$, ^{a} $p < .01$
Same results as in figures 6

E.2 German Specific Results

Table E8: Germany only - Nr. cases on LHS: Results using joint week dummy(w8+).

	Mar	Apr	May	Jun	Jul	Aug	Sep	Oct	Nov	Dec	Jan
Break =>W8	0.557^{a}	0.449^{a}	0.104	-0.219	-0.174	0.147^{c}	0.348^{a}	0.324^{a}	0.445^{a}	0.320^{b}	0.124
	(0.123)	(0.162)	(0.217)	(0.138)	(0.126)	(0.0842)	(0.103)	(0.0913)	(0.100)	(0.129)	(0.0909)
Population	0.765^{a}	0.961^{a}	1.085^{a}	1.044^{a}	1.258^{a}	0.917^{a}	1.179^{a}	1.314^{a}	1.192^{a}	1.116^{a}	1.072^{a}
	(0.178)	(0.203)	(0.253)	(0.304)	(0.252)	(0.140)	(0.132)	(0.108)	(0.107)	(0.146)	(0.132)
Median age	4.715^{b}	11.53^{a}	12.94^{a}	12.65^{a}	12.86^{a}	7.887^{a}	3.818^{c}	3.327^{c}	3.304^{b}	0.217	-1.483
	(1.765)	(2.247)	(2.863)	(2.862)	(3.110)	(1.772)	(2.090)	(1.836)	(1.564)	(2.039)	(1.671)
Share over age 60	-4.626^{a}	-7.438^{a}	-7.893^{a}	-7.241^{a}	-8.448^{a}	-6.318^{a}	-3.200^{b}	-2.196^{b}	-1.235	1.522	2.595^{b}
	(0.967)	(1.217)	(1.844)	(1.679)	(1.919)	(1.073)	(1.256)	(1.033)	(1.019)	(1.459)	(1.054)
Share below 14	-0.0610	-0.100	-0.210	0.194	0.204	1.046	1.960^{a}	0.945	1.221^{b}	0.713	0.257
	(0.662)	(0.956)	(1.467)	(0.994)	(0.987)	(0.689)	(0.641)	(0.610)	(0.584)	(0.639)	(0.488)
Area (km sq.)	0.00245	-0.226^{a}	-0.328^{a}	-0.445^{a}	-0.419^{a}	-0.299^{a}	-0.340^{a}	-0.185^{a}	-0.163^{a}	-0.0860^{c}	-0.0182
	(0.0568)	(0.0644)	(0.0869)	(0.109)	(0.102)	(0.0456)	(0.0564)	(0.0533)	(0.0467)	(0.0494)	(0.0595)
Income	0.336^{a}	0.345^{b}	0.256	0.303	0.163	0.304^{a}	0.223^{c}	0.0208	0.0513	0.0552	0.00710
	(0.117)	(0.136)	(0.204)	(0.246)	(0.175)	(0.100)	(0.123)	(0.102)	(0.0830)	(0.113)	(0.0901)
Interm. urb.	0.120	0.222^{b}	0.136	-0.0275	-0.0483	-0.124	0.0995	-0.108	-0.0979	-0.0775	-0.0320
	(0.111)	(0.105)	(0.165)	(0.181)	(0.169)	(0.0935)	(0.0828)	(0.0911)	(0.0668)	(0.0762)	(0.0804)
Rural	0.0754	0.423^{b}	0.289	0.0572	0.0201	-0.0877	0.246^{c}	-0.0562	-0.0253	0.0495	0.129
	(0.125)	(0.182)	(0.255)	(0.255)	(0.216)	(0.138)	(0.125)	(0.120)	(0.110)	(0.117)	(0.122)
Constant	-9.715^{b}	-28.14^{a}	-33.04^{a}	-34.49^{a}	-32.54^{a}	-19.71^{a}	-19.06^{a}	-16.57^{a}	-18.79^{a}	-14.11^{a}	-9.659^{a}
	(3.989)	(5.462)	(6.107)	(5.628)	(6.875)	(3.231)	(4.666)	(4.043)	(3.923)	(3.499)	(3.251)
Observations	401	401	401	401	401	401	401	401	401	401	401

Standard errors, clustered at NUTS 2 level, in parenthesis. ^{c} $p < .1$, ^{b} $p < .05$, ^{a} $p < .01$
Same results as in figure 5.

Table E9: Germany only - Nr. cases on LHS: Results using separate week specific dummies(w8, w9 and w10).

	Mar	Apr	May	Jun	Jul	Aug	Sep	Oct	Nov	Dec	Jan
Break W8	0.330^{a}	0.0672	-0.324	-0.314^{c}	-0.327^{c}	0.146	0.0344	0.314^{b}	0.364^{a}	0.229	0.0202
	(0.106)	(0.145)	(0.212)	(0.167)	(0.180)	(0.0926)	(0.0929)	(0.141)	(0.126)	(0.165)	(0.112)
Break W9/W10	0.735^{a}	0.765^{a}	0.457^{c}	-0.136	-0.0447	0.154	0.608^{a}	0.338^{a}	0.518^{a}	0.405^{a}	0.220^{b}
	(0.151)	(0.177)	(0.263)	(0.134)	(0.143)	(0.111)	(0.0926)	(0.0943)	(0.0951)	(0.124)	(0.100)
Population	0.861^{a}	0.968^{a}	1.188^{a}	1.069^{a}	1.337^{a}	0.961^{a}	1.236^{a}	1.347^{a}	1.251^{a}	1.143^{a}	1.065^{a}
	(0.178)	(0.176)	(0.214)	(0.293)	(0.227)	(0.139)	(0.145)	(0.122)	(0.0944)	(0.113)	(0.0856)
Median age	3.262^{c}	8.991^{a}	10.66^{a}	12.44^{a}	12.53^{a}	8.509^{a}	2.150	3.833^{b}	3.497^{b}	0.267	-1.707
	(1.922)	(2.443)	(2.786)	(2.860)	(2.931)	(1.758)	(1.757)	(1.835)	(1.525)	(2.044)	(1.559)
Share over age 60	-3.494^{a}	-5.649^{a}	-6.090^{a}	-6.998^{a}	-8.025^{a}	-6.607^{a}	-1.901^{c}	-2.424^{b}	-1.191	1.620	2.819^{b}
	(1.105)	(1.345)	(1.911)	(1.682)	(1.908)	(1.075)	(1.094)	(1.080)	(1.037)	(1.502)	(1.082)
Share below 14	0.227	0.134	0.102	0.207	0.287	0.994	2.157^{a}	0.891	1.220^{b}	0.677	0.209
	(0.660)	(0.918)	(1.369)	(0.963)	(0.939)	(0.640)	(0.499)	(0.571)	(0.591)	(0.661)	(0.507)
Area (km sq.)	0.00869	-0.146^{b}	-0.290^{a}	-0.442^{a}	-0.432^{a}	-0.329^{a}	-0.304^{a}	-0.207^{a}	-0.183^{a}	-0.0886^{b}	-0.000229
	(0.0483)	(0.0563)	(0.0683)	(0.0791)	(0.0810)	(0.0450)	(0.0395)	(0.0491)	(0.0369)	(0.0386)	(0.0381)
Income	0.246^{c}	0.243^{c}	0.125	0.282	0.117	0.306^{a}	0.133	0.0232	0.0310	0.0409	-0.00465
	(0.135)	(0.129)	(0.182)	(0.252)	(0.173)	(0.102)	(0.129)	(0.106)	(0.0888)	(0.105)	(0.0813)
Constant	-9.015^{b}	-24.51^{a}	-31.36^{a}	-34.66^{a}	-33.40^{a}	-21.45^{a}	-17.57^{a}	-17.96^{a}	-20.14^{a}	-14.75^{a}	-9.354^{a}
	(3.775)	(5.433)	(4.847)	(5.198)	(6.118)	(3.339)	(3.967)	(3.547)	(3.386)	(3.373)	(2.594)
Observations	401	401	401	401	401	401	401	401	401	401	401

Standard errors, clustered at NUTS 2 level, in parenthesis. ^{c} $p < .1$, ^{b} $p < .05$, ^{a} $p < .01$
Same results as in figure 5.

Table E10: Germany only - Nr. Deaths on LHS: Results using separate week specific dummies(w8, w9/w10).

	Mar	Apr	May	Jun	Jul	Aug	Sep	Oct	Nov	Dec	Jan
Break W8	-0.0371	-0.0512	-0.123	-0.110^{b}	-0.133^{a}	-0.0587	0.0633	0.295	0.514^{b}	0.194	0.0626
	(0.152)	(0.139)	(0.0998)	(0.0472)	(0.0422)	(0.0498)	(0.0863)	(0.202)	(0.198)	(0.205)	(0.148)
Break W9/W10	0.740^{a}	0.696^{a}	0.210	-0.0267	-0.0439	-0.0189	0.0642	0.315^{b}	0.603^{a}	0.419^{b}	0.177
	(0.159)	(0.159)	(0.135)	(0.0598)	(0.0363)	(0.0499)	(0.0679)	(0.129)	(0.147)	(0.158)	(0.134)
Population	0.542	0.748^{a}	0.329^{c}	0.0536	0.489^{a}	0.345^{a}	0.388^{a}	1.246^{a}	1.314^{a}	1.338^{a}	1.327^{a}
	(0.323)	(0.249)	(0.173)	(0.154)	(0.118)	(0.105)	(0.127)	(0.224)	(0.175)	(0.221)	(0.217)
Median age	3.405	8.035^{a}	5.850^{a}	3.103^{a}	2.925^{a}	2.537^{a}	3.273^{b}	3.427	0.407	0.845	-0.925
	(2.618)	(2.713)	(1.573)	(0.993)	(0.826)	(0.797)	(1.368)	(2.842)	(2.582)	(2.846)	(1.807)
Share over age 60	-2.858^{c}	-4.097^{b}	-2.068^{c}	-1.392^{b}	-1.593^{a}	-1.236^{a}	-1.252	0.252	1.381	2.260	3.617^{a}
	(1.521)	(1.524)	(1.122)	(0.594)	(0.546)	(0.441)	(0.908)	(1.801)	(1.759)	(2.036)	(1.263)
Share below 14	0.131	0.0252	-0.164	-0.471	-0.541	-0.323	0.253	2.059^{c}	1.668^{c}	0.117	0.0948
	(0.970)	(1.008)	(0.623)	(0.403)	(0.343)	(0.299)	(0.545)	(1.125)	(0.935)	(0.943)	(0.769)
Area (km sq.)	0.0161	-0.171^{b}	-0.115^{c}	-0.0916^{c}	-0.143^{a}	-0.133^{a}	-0.125^{a}	-0.234^{a}	-0.183^{b}	-0.155^{b}	-0.0761
	(0.0706)	(0.0782)	(0.0668)	(0.0485)	(0.0339)	(0.0363)	(0.0442)	(0.0750)	(0.0725)	(0.0641)	(0.0703)
Income	0.440^{c}	0.356^{c}	0.257^{b}	0.259^{b}	-0.142^{c}	-0.0550	0.137^{c}	0.0758	-0.145	-0.147	-0.208
	(0.255)	(0.188)	(0.121)	(0.102)	(0.0766)	(0.0699)	(0.0776)	(0.187)	(0.150)	(0.195)	(0.167)
Interm. urb.	0.0811	0.171	-0.0235	-0.0282	0.0134	0.00177	0.0201	-0.0854	-0.207	-0.201^{c}	-0.122
	(0.162)	(0.142)	(0.0708)	(0.0728)	(0.0491)	(0.0571)	(0.0740)	(0.151)	(0.136)	(0.117)	(0.119)
Rural	0.0221	0.358^{c}	0.0786	0.00410	0.108	0.0671	0.0730	0.0519	-0.0188	0.00505	0.200
	(0.194)	(0.210)	(0.118)	(0.105)	(0.0669)	(0.0760)	(0.0974)	(0.195)	(0.196)	(0.164)	(0.149)
Constant	-12.50^{b}	-26.79^{a}	-20.08^{a}	-8.093^{a}	-8.069^{a}	-7.441^{a}	-13.76^{a}	-31.70^{a}	-21.06^{a}	-21.27^{a}	-19.05^{a}
	(5.356)	(6.888)	(3.484)	(2.649)	(2.149)	(2.463)	(3.183)	(6.026)	(5.758)	(4.823)	(3.763)
Observations	401	401	401	401	401	401	401	401	401	401	401

Standard errors, clustered at NUTS 2 level, in parenthesis. ^{c} $p < .1$, ^{b} $p < .05$, ^{a} $p < .01$
Same results as in figure 5.

Table E11: Germany only - Nr. deaths on LHS: Results using joint week dummy(w8+).

	Mar	Apr	May	Jun	Jul	Aug	Sep	Oct	Nov	Dec	Jan
Break =>W8	0.395^{b}	0.364^{b}	0.0668	-0.0542	-0.0749^{b}	-0.0306	0.0630	0.277^{c}	0.544^{a}	0.328^{b}	0.132
	(0.161)	(0.157)	(0.107)	(0.0479)	(0.0310)	(0.0422)	(0.0657)	(0.141)	(0.153)	(0.150)	(0.109)
Population	0.296	0.505^{b}	0.217	0.0141	0.446^{a}	0.322^{a}	0.386^{a}	1.276^{a}	1.320^{a}	1.279^{a}	1.308^{a}
	(0.298)	(0.240)	(0.172)	(0.152)	(0.112)	(0.0994)	(0.129)	(0.231)	(0.184)	(0.228)	(0.220)
Median age	4.290^{c}	8.759^{a}	5.674^{a}	2.321^{b}	2.408^{a}	1.987^{b}	2.653^{b}	4.300	1.004	0.255	-0.917
	(2.370)	(2.562)	(1.482)	(0.908)	(0.754)	(0.739)	(1.249)	(2.564)	(2.301)	(2.154)	(1.455)
Share above age 60	-0.137^{a}	-0.174^{a}	-0.0743^{b}	-0.0289^{c}	-0.0399^{a}	-0.0290^{b}	-0.0361	-0.0392	0.00963	0.0827^{c}	0.122^{a}
	(0.0448)	(0.0495)	(0.0336)	(0.0147)	(0.0126)	(0.0116)	(0.0252)	(0.0536)	(0.0499)	(0.0465)	(0.0251)
Area (km sq.)	0.0479	-0.140^{c}	-0.0963	-0.0830^{c}	-0.141^{a}	-0.128^{a}	-0.109^{b}	-0.204^{b}	-0.152^{b}	-0.130^{b}	-0.0684
	(0.0763)	(0.0793)	(0.0660)	(0.0487)	(0.0352)	(0.0352)	(0.0437)	(0.0802)	(0.0723)	(0.0614)	(0.0695)
Income	0.612^{a}	0.527^{a}	0.331^{b}	0.281^{a}	-0.114	-0.0423	0.130	0.0493	-0.159	-0.121	-0.203
	(0.224)	(0.189)	(0.129)	(0.102)	(0.0716)	(0.0666)	(0.0812)	(0.198)	(0.147)	(0.203)	(0.170)
Interm. urb.	0.120	0.209	-0.0127	-0.0315	0.0162	-0.000963	0.00654	-0.0998	-0.220	-0.209^{c}	-0.124
	(0.170)	(0.152)	(0.0700)	(0.0720)	(0.0494)	(0.0550)	(0.0758)	(0.156)	(0.143)	(0.117)	(0.125)
Rural	0.0772	0.414^{c}	0.0995	0.0103	0.123^{c}	0.0705	0.0526	-0.00267	-0.0627	-0.00198	0.199
	(0.216)	(0.217)	(0.113)	(0.104)	(0.0676)	(0.0730)	(0.0994)	(0.202)	(0.200)	(0.170)	(0.155)
Constant	-19.96^{b}	-36.98^{a}	-24.03^{a}	-9.904^{a}	-11.42^{a}	-9.338^{a}	-13.90^{a}	-28.07^{a}	-14.83^{c}	-13.17^{c}	-10.08^{b}
	(7.853)	(8.589)	(5.048)	(3.403)	(2.781)	(2.792)	(4.213)	(8.498)	(7.604)	(7.070)	(4.949)
Observations	401	401	401	401	401	401	401	401	401	401	401

Standard errors, clustered at NUTS 2 level, in parenthesis. ^{c} $p < .1$, ^{b} $p < .05$, ^{a} $p < .01$
Same results as in figure 5.

F Broad Results Robustness

Table F12: Robustness results using a joint week 8+ dummy. Classification of Mecklenburg-Vorpommern changed (see appendix C)

	Mar	Apr	May	Jun	Jul	Aug	Sep	Oct	Nov	Dec	Jan
Break =>W8	0.401^{a}	0.253	-0.0571	-0.191	-0.115	0.0975	0.234^{b}	0.206^{b}	0.291^{a}	0.184	0.0454
	(0.126)	(0.170)	(0.205)	(0.129)	(0.131)	(0.0983)	(0.106)	(0.0919)	(0.108)	(0.128)	(0.0965)
Population	0.472^{a}	0.897^{a}	0.895^{a}	0.780^{a}	0.980^{a}	0.750^{a}	1.080^{a}	1.235^{a}	1.213^{a}	1.199^{a}	1.193^{a}
	(0.129)	(0.195)	(0.215)	(0.225)	(0.212)	(0.133)	(0.119)	(0.107)	(0.121)	(0.128)	(0.103)
Median age	5.837^{a}	10.25^{a}	10.04^{a}	6.630^{a}	5.373^{b}	5.778^{a}	2.251	0.271	2.592^{b}	2.456^{c}	0.431
	(1.378)	(1.580)	(1.703)	(2.410)	(2.209)	(1.381)	(1.517)	(1.284)	(1.239)	(1.395)	(1.070)
Share below 14	-0.196	-0.159	0.288	1.146^{c}	1.329^{b}	0.645	1.293^{b}	1.022^{b}	0.385	-0.204	-0.0368
	(0.543)	(0.685)	(0.911)	(0.661)	(0.646)	(0.498)	(0.510)	(0.403)	(0.441)	(0.452)	(0.342)
Share over age 60	-4.752^{a}	-6.615^{a}	-5.682^{a}	-3.359^{b}	-3.511^{b}	-4.969^{a}	-2.688^{a}	-1.190	-1.624^{c}	-0.594	0.929
	(0.847)	(1.015)	(1.152)	(1.490)	(1.479)	(0.879)	(0.908)	(0.804)	(0.925)	(1.077)	(0.744)
Area (km sq.)	0.0169	-0.184^{a}	-0.247^{a}	-0.295^{a}	-0.249^{a}	-0.199^{a}	-0.254^{a}	-0.0981^{a}	-0.120^{a}	-0.122^{a}	-0.0703^{b}
	(0.0427)	(0.0495)	(0.0602)	(0.0730)	(0.0663)	(0.0430)	(0.0387)	(0.0342)	(0.0301)	(0.0316)	(0.0347)
Income	0.567^{a}	0.346^{a}	0.326^{b}	0.419^{b}	0.278^{c}	0.414^{a}	0.181^{c}	-0.0877	-0.0794	-0.0413	-0.0488
	(0.0991)	(0.124)	(0.156)	(0.173)	(0.156)	(0.0962)	(0.0979)	(0.0870)	(0.0844)	(0.0907)	(0.0712)
Interm. urb.	0.0136	0.0944	-0.0480	-0.160	-0.234^{c}	-0.171^{b}	-0.0290	-0.195^{a}	-0.134^{b}	-0.0169	0.0291
	(0.0877)	(0.0995)	(0.133)	(0.144)	(0.140)	(0.0788)	(0.0661)	(0.0685)	(0.0567)	(0.0628)	(0.0562)
Rural	-0.0132	0.319^{c}	0.0693	-0.0794	-0.128	-0.212^{c}	0.0114	-0.144	-0.0807	0.0386	0.133^{c}
	(0.112)	(0.163)	(0.206)	(0.191)	(0.171)	(0.120)	(0.108)	(0.0995)	(0.0916)	(0.0904)	(0.0788)
BE	0.310	2.093^{a}	2.561^{a}	0.916^{a}	0.471^{c}	0.274	-0.139	0.740^{a}	-1.143^{a}	-0.704^{a}	-0.372^{b}
	(0.244)	(0.273)	(0.370)	(0.337)	(0.268)	(0.176)	(0.217)	(0.210)	(0.187)	(0.191)	(0.158)
DE	0.0134	0.895^{a}	1.148^{a}	-0.0302	-0.631^{a}	-0.176^{c}	-1.098^{a}	-1.028^{a}	-1.387^{a}	-0.384^{a}	-0.320^{a}
	(0.194)	(0.203)	(0.273)	(0.201)	(0.168)	(0.0998)	(0.160)	(0.0946)	(0.120)	(0.122)	(0.117)
DK	-0.225	1.181^{a}	1.720^{a}	0.461	-0.356	-0.115	-0.0222	-1.125^{a}	-1.348^{a}	0.324^{c}	-0.448^{a}
	(0.248)	(0.312)	(0.404)	(0.404)	(0.317)	(0.357)	(0.204)	(0.140)	(0.175)	(0.182)	(0.141)
EE	-0.935^{a}	0.743^{b}	1.722^{a}	0.439	-0.980^{a}	-0.337	-0.374	-2.296^{a}	-1.546^{a}	0.135	0.547^{a}
	(0.268)	(0.317)	(0.370)	(0.371)	(0.346)	(0.214)	(0.266)	(0.192)	(0.208)	(0.238)	(0.174)
FI	-1.153^{a}	0.560	1.552^{a}	-0.248	-1.212^{a}	-0.850^{a}	-1.230^{a}	-2.411^{a}	-2.903^{a}	-2.119^{a}	-1.973^{a}
	(0.298)	(0.340)	(0.448)	(0.430)	(0.392)	(0.220)	(0.309)	(0.203)	(0.264)	(0.326)	(0.233)
IE	-0.995^{a}	2.103^{a}	2.714^{a}	0.263	-1.025^{a}	-0.588^{b}	-1.042^{a}	-1.354^{a}	-2.733^{a}	-0.567^{b}	1.374^{a}
	(0.311)	(0.396)	(0.582)	(0.418)	(0.326)	(0.253)	(0.319)	(0.267)	(0.243)	(0.254)	(0.204)
LV	-1.267^{a}	0.107	1.483^{a}	0.114	-1.077^{a}	-0.626^{a}	-1.884^{a}	-1.390^{a}	-1.244^{a}	0.366^{c}	0.737^{a}
	(0.222)	(0.256)	(0.368)	(0.325)	(0.306)	(0.187)	(0.229)	(0.164)	(0.180)	(0.194)	(0.164)
NL	-0.952^{a}	1.676^{a}	1.535^{a}	0.263	-0.883^{a}	-0.0556	-0.230	-0.149	-1.333^{a}	0.300	0.356^{b}
	(0.273)	(0.294)	(0.396)	(0.355)	(0.313)	(0.196)	(0.213)	(0.155)	(0.179)	(0.191)	(0.149)
NO	-1.605^{a}	0.186	0.915^{c}	-0.0303	-1.466^{a}	-0.716^{a}	-1.558^{a}	-2.322^{a}	-2.388^{a}	-1.630^{a}	-1.193^{a}
	(0.300)	(0.303)	(0.471)	(0.370)	(0.366)	(0.271)	(0.258)	(0.208)	(0.237)	(0.357)	(0.293)
PT	-0.991^{b}	1.320^{a}	2.448^{a}	1.959^{a}	-0.0234				-3.081^{a}	-1.892^{a}	-0.797^{a}
	(0.475)	(0.408)	(0.389)	(0.460)	(0.389)				(0.305)	(0.258)	(0.149)
SE	-0.527	2.706^{a}	4.823^{a}	4.327^{a}	1.695^{a}	1.102^{a}	-0.265	-0.937^{a}	-0.354	0.844^{a}	0.518^{b}
	(0.346)	(0.398)	(0.448)	(0.459)	(0.443)	(0.320)	(0.299)	(0.221)	(0.254)	(0.278)	(0.215)
SI	-0.740^{b}	-0.292	-0.136	1.194^{a}	0.467^{c}	0.370^{c}	0.0105	0.368^{b}	-0.175	0.835^{a}	1.149^{a}
	(0.308)	(0.364)	(0.330)	(0.311)	(0.275)	(0.208)	(0.275)	(0.142)	(0.191)	(0.222)	(0.139)
SK	-2.348^{a}	-0.458	-0.288	0.0943	-0.452	-0.268	-0.374	-0.370^{c}	-1.237^{a}	0.422^{b}	0.697^{a}
	(0.281)	(0.341)	(0.419)	(0.455)	(0.298)	(0.288)	(0.289)	(0.198)	(0.216)	(0.210)	(0.182)
Constant	-11.71^{a}	-26.07^{a}	-30.38^{a}	-25.46^{a}	-21.20^{a}	-14.44^{a}	-10.79^{a}	-5.953^{b}	-10.54^{a}	-12.80^{a}	-11.01^{a}
	(3.341)	(4.147)	(4.961)	(4.628)	(4.640)	(2.895)	(3.518)	(2.810)	(2.560)	(2.141)	(2.158)
Observations	648	650	650	650	650	625	625	625	650	650	650

Standard errors, clustered at NUTS 2 level, in parenthesis. ^{c} $p < .1$, ^{b} $p < .05$, ^{a} $p < .01$

Table F13: Robustness after changing classification of Mecklenburg-Vorpommern. Separate week specific dummies(w8, w9 and w10)

	Mar	Apr	May	Jun	Jul	Aug	Sep	Oct	Nov	Dec	Jan
Break W8	0.178	-0.0856	-0.443^b	-0.329^b	-0.280^c	0.00836	-0.0208	0.144	0.152	0.113	0.0232
	(0.119)	(0.165)	(0.176)	(0.149)	(0.149)	(0.113)	(0.0901)	(0.112)	(0.129)	(0.148)	(0.105)
Break W9	0.626^a	0.594^a	0.342	-0.0526	0.0551	0.188^c	0.502^a	0.277^a	0.433^a	0.264^b	0.0712
	(0.124)	(0.149)	(0.222)	(0.130)	(0.130)	(0.109)	(0.0868)	(0.0835)	(0.0883)	(0.114)	(0.102)
Break W10	0.414^b	0.574^a	-0.143	-0.0872	-0.175	0.179	0.0963	-0.0836	0.328^b	-0.153	-0.0867
	(0.175)	(0.215)	(0.302)	(0.273)	(0.218)	(0.198)	(0.180)	(0.108)	(0.159)	(0.166)	(0.125)
Population	0.562^a	1.107^a	1.113^a	0.864^a	1.072^a	0.807^a	1.222^a	1.255^a	1.295^a	1.224^a	1.200^a
	(0.135)	(0.173)	(0.189)	(0.232)	(0.212)	(0.129)	(0.108)	(0.102)	(0.108)	(0.113)	(0.0949)
Median age	5.018^a	9.144^a	8.451^a	6.164^b	4.678^b	5.454^a	1.015	-0.245	2.070	1.928	0.246
	(1.414)	(1.606)	(1.755)	(2.445)	(2.212)	(1.408)	(1.534)	(1.317)	(1.254)	(1.379)	(1.077)
Share below 14	0.0815	0.263	0.745	1.316^b	1.522^b	0.766	1.624^a	1.090^a	0.553	-0.137	-0.0173
	(0.516)	(0.609)	(0.819)	(0.646)	(0.627)	(0.487)	(0.470)	(0.406)	(0.438)	(0.449)	(0.339)
Share over age 60	-3.978^a	-5.503^a	-4.290^a	-2.900^c	-2.912^b	-4.646^a	-1.639^c	-0.846	-1.142	-0.250	1.044
	(0.876)	(0.989)	(1.130)	(1.492)	(1.459)	(0.861)	(0.899)	(0.820)	(0.926)	(1.060)	(0.762)
Area (km sq.)	0.00897	-0.220^a	-0.266^a	-0.308^a	-0.256^a	-0.209^a	-0.263^a	-0.0875^b	-0.130^a	-0.111^a	-0.0655^c
	(0.0435)	(0.0448)	(0.0587)	(0.0754)	(0.0690)	(0.0429)	(0.0364)	(0.0362)	(0.0300)	(0.0316)	(0.0350)
Income	0.501^a	0.196^c	0.162	0.359^b	0.208	0.374^a	0.0768	-0.107	-0.139^c	-0.0659	-0.0560
	(0.105)	(0.116)	(0.140)	(0.178)	(0.157)	(0.0968)	(0.0934)	(0.0843)	(0.0802)	(0.0828)	(0.0673)
Interm. urb.	0.000980	0.0963	-0.0664	-0.161	-0.243^c	-0.171^b	-0.0463	-0.212^a	-0.138^b	-0.0350	0.0222
	(0.0839)	(0.0948)	(0.125)	(0.144)	(0.134)	(0.0788)	(0.0648)	(0.0682)	(0.0553)	(0.0622)	(0.0569)
Rural	-0.0459	0.300^c	0.0257	-0.0881	-0.148	-0.215^c	-0.0189	-0.165	-0.0930	0.0146	0.124
	(0.102)	(0.152)	(0.192)	(0.194)	(0.163)	(0.118)	(0.102)	(0.0998)	(0.0867)	(0.0888)	(0.0797)
BE	-0.0878	1.463^a	1.846^a	0.660^c	0.167	0.105	-0.620^a	0.626^a	-1.401^a	-0.832^a	-0.412^b
	(0.240)	(0.249)	(0.389)	(0.350)	(0.282)	(0.198)	(0.196)	(0.212)	(0.183)	(0.184)	(0.168)
DE	-0.120	0.667^a	0.910^a	-0.122	-0.731^a	-0.237^b	-1.253^a	-1.053^a	-1.476^a	-0.412^a	-0.328^a
	(0.179)	(0.181)	(0.236)	(0.208)	(0.189)	(0.104)	(0.133)	(0.101)	(0.128)	(0.128)	(0.120)
DK	-0.369	0.960^a	1.467^a	0.371	-0.464	-0.177	-0.202	-1.170^a	-1.439^a	0.277	-0.463^a
	(0.224)	(0.278)	(0.370)	(0.410)	(0.335)	(0.363)	(0.189)	(0.141)	(0.169)	(0.182)	(0.142)
EE	-1.387^a	0.0382	0.879^b	0.151	-1.341^a	-0.526^b	-0.951^a	-2.460^a	-1.843^a	-0.0476	0.487^a
	(0.262)	(0.283)	(0.370)	(0.383)	(0.352)	(0.226)	(0.246)	(0.195)	(0.193)	(0.217)	(0.183)
FI	-1.434^a	0.0967	1.094^b	-0.432	-1.404^a	-0.977^a	-1.542^a	-2.452^a	-3.078^a	-2.155^a	-1.980^a
	(0.287)	(0.317)	(0.477)	(0.438)	(0.383)	(0.210)	(0.303)	(0.190)	(0.263)	(0.302)	(0.237)
IE	-0.885^a	2.270^a	2.887^a	0.330	-0.952^a	-0.546^b	-0.937^a	-1.337^a	-2.668^a	-0.547^b	1.379^a
	(0.285)	(0.354)	(0.520)	(0.415)	(0.344)	(0.253)	(0.271)	(0.256)	(0.238)	(0.256)	(0.209)
LV	-1.496^a	-0.279	1.026^a	-0.0433	-1.273^a	-0.730^a	-2.196^a	-1.476^a	-1.406^a	0.269	0.705^a
	(0.209)	(0.223)	(0.319)	(0.332)	(0.310)	(0.188)	(0.208)	(0.169)	(0.180)	(0.185)	(0.162)
NL	-1.101^a	1.386^a	1.217^a	0.146	-1.018^a	-0.135	-0.447^b	-0.194	-1.449^a	0.250	0.341^b
	(0.234)	(0.243)	(0.346)	(0.339)	(0.300)	(0.192)	(0.195)	(0.145)	(0.166)	(0.184)	(0.148)
NO	-1.601^a	0.213	0.951^b	-0.0192	-1.451^a	-0.711^a	-1.544^a	-2.319^a	-2.376^a	-1.620^a	-1.190^a
	(0.284)	(0.279)	(0.446)	(0.376)	(0.370)	(0.265)	(0.226)	(0.205)	(0.252)	(0.357)	(0.293)
PT	-1.384^a	0.686^c	1.711^a	1.701^a	-0.338				-3.345^a	-2.038^a	-0.843^a
	(0.479)	(0.385)	(0.382)	(0.473)	(0.406)				(0.303)	(0.249)	(0.159)
SE	-0.717^b	2.409^a	4.517^a	4.208^a	1.566^a	1.019^a	-0.482^c	-0.976^a	-0.469^c	0.811^a	0.510^b
	(0.339)	(0.375)	(0.420)	(0.450)	(0.430)	(0.312)	(0.268)	(0.220)	(0.254)	(0.278)	(0.220)
SI	-0.975^a	-0.687^a	-0.587^c	1.034^a	0.275	0.264	-0.293^c	0.294^b	-0.337^b	0.751^a	1.123^a
	(0.219)	(0.223)	(0.327)	(0.363)	(0.249)	(0.178)	(0.173)	(0.134)	(0.145)	(0.186)	(0.140)
SK	-2.501^a	-0.812^b	-0.593	-0.0439	-0.577^c	-0.362	-0.558^c	-0.357^b	-1.362^a	0.436^b	0.708^a
	(0.280)	(0.339)	(0.443)	(0.442)	(0.319)	(0.318)	(0.290)	(0.177)	(0.216)	(0.199)	(0.182)
Constant	-12.20^a	-27.44^a	-30.98^a	-25.96^a	-21.41^a	-14.82^a	-11.00^a	-5.403^c	-10.90^a	-12.23^a	-10.77^a
	(3.153)	(3.795)	(4.609)	(4.757)	(4.785)	(2.922)	(3.446)	(2.854)	(2.492)	(2.116)	(2.135)
Observations	648	650	650	650	650	625	625	625	650	650	650

Standard errors, clustered at NUTS 2 level, in parenthesis. ^{c} $p < .1$, ^{b} $p < .05$, ^{a} $p < .01$

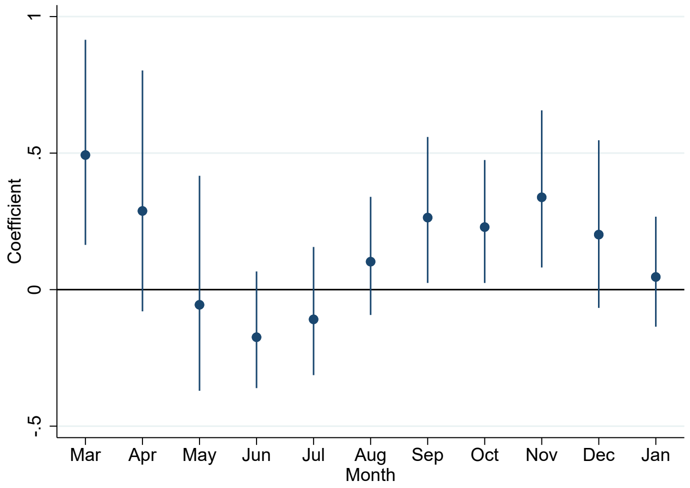

(a) Cases: Joint dummy (8, 9 or 10)

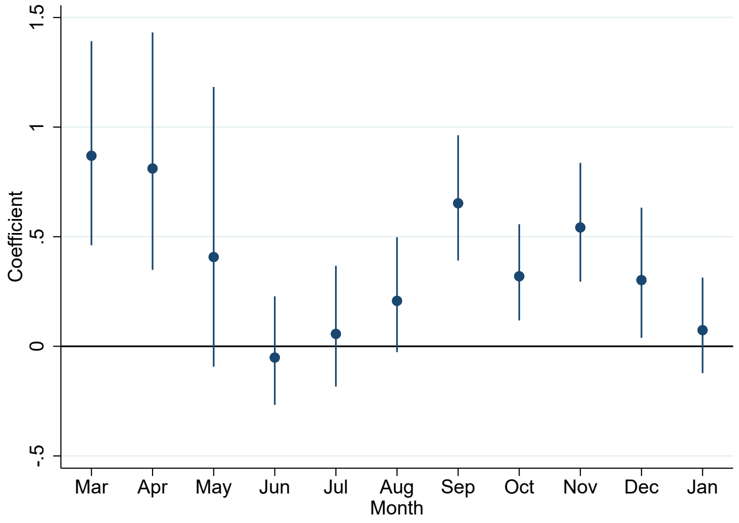

(b) Cases: Week 9 dummy

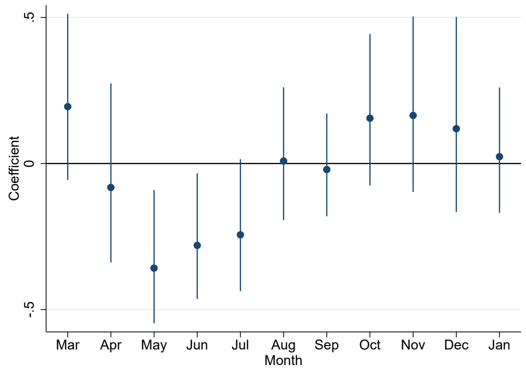

(c) Cases: Week 8 dummy

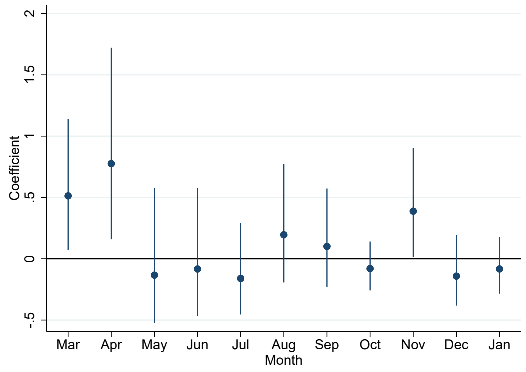

(d) Cases: Week 10 dummy

Figure F1: Robustness after changing classification in Mecklenburg-Vorpommern. Coefficient plot of the joint dummy per month in graph F1a. Sub-graphs F1c,F1b F1d show week 8, 9 and 10 dummies (in a single regression without the joint dummy).

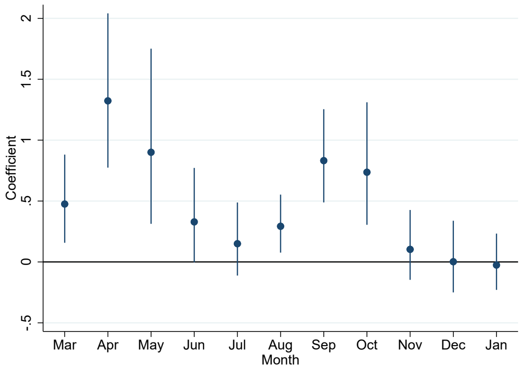

(a) Cases: Week 9 dummy (without distance)

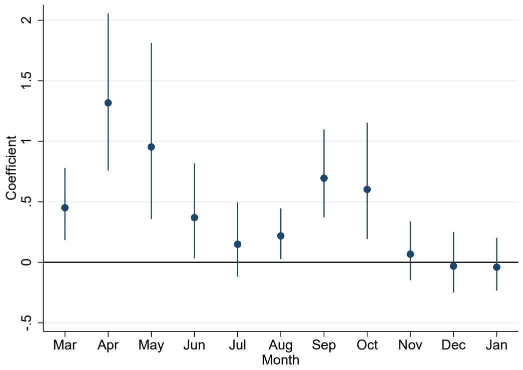

(b) Cases: Week 9 dummy (with distance)

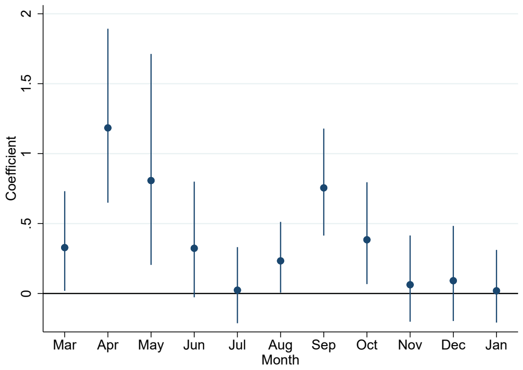

(c) Cases: Week 9 dummy (without distance)

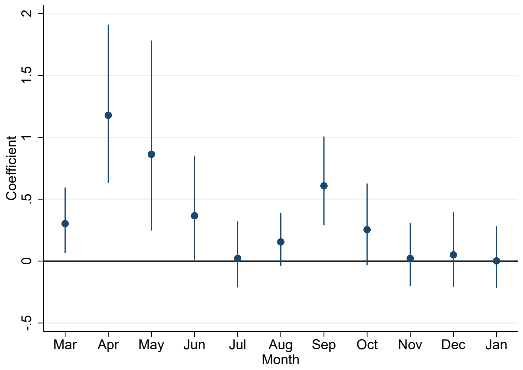

(d) Cases: Week 9 dummy (with distance)

Figure F2: Gravity robustness without country fixed effect. Comparison with/without distance to Ischgl. Coefficient plot of the week 9 dummy only from a regression as in equation 2. Lower panel (c,d) excludes Belgium. Full results tables available on request.

Table F14: Gravity robustness results using a joint late dummy. Distance from NUTS 3 region to Ischgl added.

	Mar	Apr	May	Jun	Jul	Aug	Sep	Oct	Nov	Dec	Jan
Break =>W8	0.291^{a}	0.195^{c}	-0.0400	-0.118	-0.122	0.0912	0.175^{b}	0.214^{a}	0.293^{a}	0.145	0.0324
	(0.0848)	(0.115)	(0.181)	(0.124)	(0.132)	(0.0830)	(0.0850)	(0.0771)	(0.0801)	(0.114)	(0.0927)
Distance	-0.474^{a}	-0.528^{a}	-0.257	0.0720	-0.0632	-0.206^{b}	-0.345^{a}	-0.146^{b}	-0.219^{b}	-0.296^{a}	-0.128^{c}
	(0.0852)	(0.0945)	(0.155)	(0.107)	(0.0805)	(0.0845)	(0.0776)	(0.0589)	(0.0905)	(0.0969)	(0.0715)
Population	0.658^{a}	1.219^{a}	1.045^{a}	0.723^{a}	0.999^{a}	0.888^{a}	1.300^{a}	1.343^{a}	1.356^{a}	1.380^{a}	1.269^{a}
	(0.134)	(0.157)	(0.198)	(0.233)	(0.212)	(0.131)	(0.0978)	(0.0922)	(0.103)	(0.122)	(0.109)
Median age	5.695^{a}	9.720^{a}	10.19^{a}	7.746^{a}	5.971^{b}	5.258^{a}	1.466	-0.148	2.671^{b}	2.195	0.389
	(1.257)	(1.388)	(1.580)	(2.321)	(2.282)	(1.393)	(1.454)	(1.256)	(1.070)	(1.363)	(1.081)
Share below 14	-0.409	-0.354	0.178	1.110^{c}	1.254^{c}	0.589	1.155^{b}	0.992^{b}	0.286	-0.329	-0.0899
	(0.474)	(0.617)	(0.867)	(0.647)	(0.651)	(0.465)	(0.458)	(0.389)	(0.428)	(0.463)	(0.351)
Share over age 60	-4.432^{a}	-5.990^{a}	-5.704^{a}	-4.328^{a}	-4.041^{a}	-4.442^{a}	-1.886^{b}	-0.767	-1.613^{b}	-0.249	1.001
	(0.690)	(0.822)	(1.090)	(1.403)	(1.533)	(0.884)	(0.847)	(0.767)	(0.781)	(1.068)	(0.768)
Area (km sq.)	0.0106	-0.209^{a}	-0.257^{a}	-0.286^{a}	-0.248^{a}	-0.210^{a}	-0.274^{a}	-0.106^{a}	-0.129^{a}	-0.137^{a}	-0.0759^{b}
	(0.0409)	(0.0473)	(0.0622)	(0.0748)	(0.0669)	(0.0436)	(0.0378)	(0.0358)	(0.0317)	(0.0321)	(0.0351)
Income	0.417^{a}	0.105	0.199	0.425^{b}	0.237	0.322^{a}	0.0329	-0.157^{b}	-0.195^{a}	-0.175^{b}	-0.109
	(0.104)	(0.110)	(0.154)	(0.180)	(0.157)	(0.0953)	(0.0863)	(0.0772)	(0.0737)	(0.0870)	(0.0747)
Interm. urb.	-0.0275	0.0644	-0.0879	-0.205	-0.258^{c}	-0.179^{b}	-0.0393	-0.199^{a}	-0.157^{a}	-0.0379	0.0192
	(0.0741)	(0.0913)	(0.135)	(0.140)	(0.141)	(0.0759)	(0.0610)	(0.0676)	(0.0520)	(0.0571)	(0.0558)
Rural	-0.0828	0.275^{c}	-0.00565	-0.183	-0.193	-0.210^{c}	0.0226	-0.136	-0.121	0.0139	0.114
	(0.0971)	(0.154)	(0.212)	(0.182)	(0.168)	(0.117)	(0.0994)	(0.0977)	(0.0834)	(0.0835)	(0.0785)
BE	0.855^{a}	2.609^{a}	2.781^{a}	0.813^{b}	0.545^{c}	0.457^{b}	0.213	0.859^{a}	-0.942^{a}	-0.407^{b}	-0.247
	(0.214)	(0.257)	(0.405)	(0.364)	(0.284)	(0.182)	(0.221)	(0.217)	(0.200)	(0.200)	(0.166)
DE	0.196	1.067^{a}	1.226^{a}	-0.0504	-0.601^{a}	-0.117	-0.993^{a}	-0.985^{a}	-1.309^{a}	-0.289^{a}	-0.278^{a}
	(0.135)	(0.196)	(0.299)	(0.196)	(0.176)	(0.0991)	(0.166)	(0.0899)	(0.0990)	(0.100)	(0.106)
DK	0.451^{c}	1.900^{a}	2.097^{a}	0.443	-0.214	0.140	0.419^{c}	-0.950^{a}	-1.029^{a}	0.730^{a}	-0.267
	(0.238)	(0.324)	(0.458)	(0.429)	(0.341)	(0.360)	(0.222)	(0.145)	(0.203)	(0.207)	(0.161)
EE	-0.0721	1.571^{a}	2.107^{a}	0.321	-0.844^{b}	-0.0455	0.165	-2.109^{a}	-1.217^{a}	0.609^{b}	0.751^{a}
	(0.255)	(0.313)	(0.474)	(0.436)	(0.366)	(0.244)	(0.289)	(0.226)	(0.259)	(0.275)	(0.209)
FI	-0.0932	1.678^{a}	2.113^{a}	-0.312	-0.986^{b}	-0.468^{c}	-0.538	-2.158^{a}	-2.420^{a}	-1.484^{a}	-1.690^{a}
	(0.307)	(0.341)	(0.538)	(0.510)	(0.416)	(0.275)	(0.341)	(0.219)	(0.311)	(0.369)	(0.276)
IE	0.0875	3.236^{a}	3.221^{a}	0.0313	-0.911^{b}	-0.146	-0.253	-1.053^{a}	-2.295^{a}	0.0839	1.644^{a}
	(0.312)	(0.389)	(0.682)	(0.527)	(0.379)	(0.289)	(0.346)	(0.291)	(0.308)	(0.319)	(0.265)
LV	-0.628^{a}	0.734^{b}	1.804^{a}	0.0594	-0.983^{a}	-0.390^{c}	-1.484^{a}	-1.230^{a}	-0.984^{a}	0.721^{a}	0.891^{a}
	(0.222)	(0.300)	(0.460)	(0.372)	(0.313)	(0.206)	(0.256)	(0.198)	(0.226)	(0.219)	(0.182)
NL	-0.354	2.191^{a}	1.751^{a}	0.156	-0.805^{b}	0.123	0.119	-0.0334	-1.134^{a}	0.594^{a}	0.482^{a}
	(0.244)	(0.269)	(0.429)	(0.384)	(0.329)	(0.205)	(0.217)	(0.154)	(0.184)	(0.196)	(0.156)
NO	-0.516^{c}	1.382^{a}	1.474^{b}	-0.209	-1.306^{a}	-0.268	-0.762^{a}	-2.015^{a}	-1.899^{a}	-0.947^{b}	-0.902^{a}
	(0.307)	(0.316)	(0.592)	(0.476)	(0.405)	(0.319)	(0.290)	(0.248)	(0.304)	(0.394)	(0.333)
PT	0.0874	2.298^{a}	3.030^{a}	2.148^{a}	0.335				-2.582^{a}	-1.346^{a}	-0.528^{a}
	(0.416)	(0.377)	(0.452)	(0.475)	(0.407)				(0.278)	(0.268)	(0.165)
SE	0.383	3.661^{a}	5.301^{a}	4.276^{a}	1.892^{a}	1.426^{a}	0.331	-0.724^{a}	0.0609	1.391^{a}	0.760^{a}
	(0.341)	(0.396)	(0.515)	(0.529)	(0.455)	(0.340)	(0.311)	(0.234)	(0.296)	(0.321)	(0.253)
SI	-0.453	-0.0835	-0.0556	1.144^{a}	0.511^{c}	0.430^{b}	0.152	0.398^{a}	-0.105	0.958^{a}	1.201^{a}
	(0.278)	(0.347)	(0.348)	(0.316)	(0.283)	(0.203)	(0.272)	(0.137)	(0.178)	(0.212)	(0.132)
SK	-1.936^{a}	-0.144	-0.172	-0.0121	-0.419	-0.159	-0.143	-0.309	-1.142^{a}	0.607^{a}	0.772^{a}
	(0.241)	(0.321)	(0.453)	(0.468)	(0.307)	(0.276)	(0.269)	(0.195)	(0.201)	(0.197)	(0.181)
Constant	-9.740^{a}	-24.11^{a}	-29.70^{a}	-26.24^{a}	-21.02^{a}	-13.62^{a}	-9.282^{a}	-5.464^{c}	-9.977^{a}	-11.78^{a}	-10.54^{a}
	(3.118)	(3.879)	(4.770)	(4.588)	(4.858)	(2.879)	(3.341)	(2.767)	(2.484)	(2.182)	(2.188)
Observations	646	648	648	648	648	625	625	625	648	648	648

Standard errors, clustered at NUTS 2 level, in parenthesis. ^{c} $p < .1$, ^{b} $p < .05$, ^{a} $p < .01$

Table F15: Gravity robustness results using a joint late dummy. Separate week specific dummies(w8, w9 and w10)

	Mar	Apr	May	Jun	Jul	Aug	Sep	Oct	Nov	Dec	Jan
Break W8	0.186^{b}	-0.00359	-0.336^{b}	-0.237^{c}	-0.262^{c}	0.0600	-0.00193	0.203^{b}	0.232^{b}	0.158	0.0493
	(0.0838)	(0.106)	(0.155)	(0.142)	(0.156)	(0.0943)	(0.0767)	(0.0989)	(0.0937)	(0.132)	(0.100)
Break W9	0.404^{a}	0.399^{a}	0.313	0.0181	0.0462	0.119	0.388^{a}	0.242^{a}	0.358^{a}	0.143	0.0172
	(0.109)	(0.131)	(0.207)	(0.131)	(0.130)	(0.103)	(0.0848)	(0.0730)	(0.0784)	(0.113)	(0.105)
Break W10	0.488^{a}	0.718^{a}	-0.0422	-0.0455	-0.158	0.249	0.162	-0.0260	0.412^{a}	-0.0703	-0.0461
	(0.135)	(0.178)	(0.307)	(0.267)	(0.215)	(0.181)	(0.168)	(0.106)	(0.145)	(0.168)	(0.118)
Distance	-0.442^{a}	-0.479^{a}	-0.140	0.114	-0.00630	-0.203^{b}	-0.275^{a}	-0.128^{b}	-0.201^{b}	-0.289^{a}	-0.130^{c}
	(0.0847)	(0.0968)	(0.145)	(0.113)	(0.0671)	(0.0905)	(0.0651)	(0.0620)	(0.0958)	(0.101)	(0.0756)
Population	0.691^{a}	1.320^{a}	1.144^{a}	0.767^{a}	1.043^{a}	0.910^{a}	1.358^{a}	1.328^{a}	1.384^{a}	1.361^{a}	1.258^{a}
	(0.137)	(0.151)	(0.197)	(0.237)	(0.215)	(0.130)	(0.0953)	(0.0953)	(0.102)	(0.116)	(0.107)
Median age	5.494^{a}	9.406^{a}	9.141^{a}	7.382^{a}	5.451^{b}	5.273^{a}	0.789	-0.390	2.543^{b}	2.080	0.390
	(1.287)	(1.428)	(1.622)	(2.363)	(2.258)	(1.414)	(1.470)	(1.309)	(1.098)	(1.365)	(1.089)
Share below 14	-0.251	-0.0638	0.608	1.282^{b}	1.457^{b}	0.638	1.439^{a}	1.011^{b}	0.375	-0.348	-0.115
	(0.465)	(0.571)	(0.790)	(0.640)	(0.625)	(0.458)	(0.443)	(0.390)	(0.425)	(0.459)	(0.351)
Share over age 60	-4.177^{a}	-5.532^{a}	-4.820^{a}	-3.994^{a}	-3.616^{b}	-4.393^{a}	-1.316	-0.655	-1.461^{c}	-0.231	0.971
	(0.725)	(0.855)	(1.098)	(1.408)	(1.492)	(0.875)	(0.850)	(0.787)	(0.795)	(1.053)	(0.777)
Area (km sq.)	-0.000780	-0.243^{a}	-0.268^{a}	-0.294^{a}	-0.251^{a}	-0.220^{a}	-0.280^{a}	-0.0940^{b}	-0.138^{a}	-0.126^{a}	-0.0714^{c}
	(0.0417)	(0.0426)	(0.0635)	(0.0776)	(0.0699)	(0.0437)	(0.0361)	(0.0388)	(0.0317)	(0.0335)	(0.0364)
Income	0.394^{a}	0.0347	0.118	0.390^{b}	0.200	0.308^{a}	-0.0134	-0.151^{c}	-0.215^{a}	-0.165^{c}	-0.102
	(0.105)	(0.105)	(0.147)	(0.182)	(0.160)	(0.0953)	(0.0822)	(0.0778)	(0.0727)	(0.0836)	(0.0733)
Interm. urb.	-0.0251	0.0790	-0.102	-0.207	-0.266^{c}	-0.173^{b}	-0.0488	-0.211^{a}	-0.154^{a}	-0.0471	0.0164
	(0.0726)	(0.0873)	(0.128)	(0.140)	(0.135)	(0.0760)	(0.0609)	(0.0688)	(0.0510)	(0.0566)	(0.0563)
Rural	-0.0932	0.271^{c}	-0.0540	-0.199	-0.218	-0.206^{c}	-0.00679	-0.152	-0.125	0.00433	0.113
	(0.0927)	(0.151)	(0.208)	(0.187)	(0.160)	(0.117)	(0.0986)	(0.0996)	(0.0824)	(0.0852)	(0.0801)
BE	0.622^{a}	2.176^{a}	2.064^{a}	0.533	0.204	0.395^{c}	-0.223	0.810^{a}	-1.080^{a}	-0.396^{c}	-0.213
	(0.232)	(0.263)	(0.414)	(0.395)	(0.284)	(0.219)	(0.227)	(0.232)	(0.221)	(0.210)	(0.192)
DE	0.108	0.894^{a}	0.983^{a}	-0.149	-0.714^{a}	-0.146	-1.138^{a}	-0.988^{a}	-1.361^{a}	-0.274^{a}	-0.262^{b}
	(0.132)	(0.183)	(0.262)	(0.197)	(0.190)	(0.104)	(0.149)	(0.0965)	(0.106)	(0.103)	(0.112)
DK	0.340	1.705^{a}	1.738^{a}	0.306	-0.386	0.116	0.196	-0.986^{a}	-1.092^{a}	0.727^{a}	-0.253
	(0.227)	(0.303)	(0.423)	(0.441)	(0.352)	(0.369)	(0.216)	(0.157)	(0.213)	(0.216)	(0.171)
EE	-0.329	1.105^{a}	1.232^{a}	-0.0113	-1.264^{a}	-0.102	-0.367	-2.198^{a}	-1.370^{a}	0.597^{b}	0.783^{a}
	(0.285)	(0.332)	(0.470)	(0.483)	(0.356)	(0.292)	(0.300)	(0.261)	(0.299)	(0.287)	(0.240)
FI	-0.322	1.254^{a}	1.487^{a}	-0.562	-1.280^{a}	-0.534^{c}	-0.923^{a}	-2.183^{a}	-2.550^{a}	-1.455^{a}	-1.654^{a}
	(0.308)	(0.341)	(0.543)	(0.520)	(0.395)	(0.288)	(0.344)	(0.228)	(0.333)	(0.366)	(0.294)
IE	0.0769	3.241^{a}	3.101^{a}	-0.00475	-0.974^{b}	-0.134	-0.336	-1.097^{a}	-2.300^{a}	0.0537	1.638^{a}
	(0.299)	(0.362)	(0.640)	(0.536)	(0.385)	(0.300)	(0.316)	(0.290)	(0.311)	(0.324)	(0.269)
LV	-0.772^{a}	0.462	1.298^{a}	-0.133	-1.226^{a}	-0.424^{c}	-1.792^{a}	-1.280^{a}	-1.073^{a}	0.714^{a}	0.910^{a}
	(0.224)	(0.291)	(0.415)	(0.399)	(0.308)	(0.224)	(0.250)	(0.222)	(0.253)	(0.225)	(0.190)
NL	-0.469^{b}	1.958^{a}	1.362^{a}	0.00522	-0.990^{a}	0.0898	-0.120	-0.0610	-1.207^{a}	0.599^{a}	0.500^{a}
	(0.227)	(0.248)	(0.383)	(0.379)	(0.304)	(0.217)	(0.212)	(0.154)	(0.189)	(0.194)	(0.166)
NO	-0.588^{c}	1.281^{a}	1.228^{b}	-0.298	-1.427^{a}	-0.274	-0.919^{a}	-2.057^{a}	-1.935^{a}	-0.963^{b}	-0.898^{a}
	(0.298)	(0.296)	(0.565)	(0.490)	(0.408)	(0.327)	(0.270)	(0.251)	(0.316)	(0.396)	(0.339)
PT	-0.151	1.857^{a}	2.245^{a}	1.846^{a}	-0.0407				-2.725^{a}	-1.347^{a}	-0.496^{b}
	(0.438)	(0.398)	(0.438)	(0.504)	(0.413)				(0.310)	(0.279)	(0.198)
SE	0.223	3.376^{a}	4.836^{a}	4.094^{a}	1.671^{a}	1.385^{a}	0.0411	-0.757^{a}	-0.0291	1.400^{a}	0.782^{a}
	(0.354)	(0.397)	(0.486)	(0.535)	(0.436)	(0.346)	(0.296)	(0.246)	(0.308)	(0.324)	(0.264)
SI	-0.582^{b}	-0.335	-0.476	0.980^{a}	0.311	0.394^{c}	-0.102	0.369^{b}	-0.185	0.964^{a}	1.220^{a}
	(0.236)	(0.264)	(0.330)	(0.368)	(0.252)	(0.203)	(0.198)	(0.141)	(0.168)	(0.214)	(0.145)
SK	-2.074^{a}	-0.453	-0.507	-0.158	-0.572^{c}	-0.221	-0.343	-0.282	-1.231^{a}	0.656^{a}	0.803^{a}
	(0.247)	(0.322)	(0.466)	(0.459)	(0.325)	(0.296)	(0.285)	(0.193)	(0.217)	(0.189)	(0.177)
Constant	-10.47^{a}	-25.74^{a}	-30.66^{a}	-26.76^{a}	-21.41^{a}	-14.06^{a}	-9.885^{a}	-5.007^{c}	-10.40^{a}	-11.33^{a}	-10.34^{a}
	(3.051)	(3.610)	(4.470)	(4.687)	(4.916)	(2.911)	(3.323)	(2.828)	(2.430)	(2.181)	(2.179)
Observations	646	648	648	648	648	625	625	625	648	648	648

Standard errors, clustered at NUTS 2 level, in parenthesis. ^{c} $p < .1$, ^{b} $p < .05$, ^{a} $p < .01$

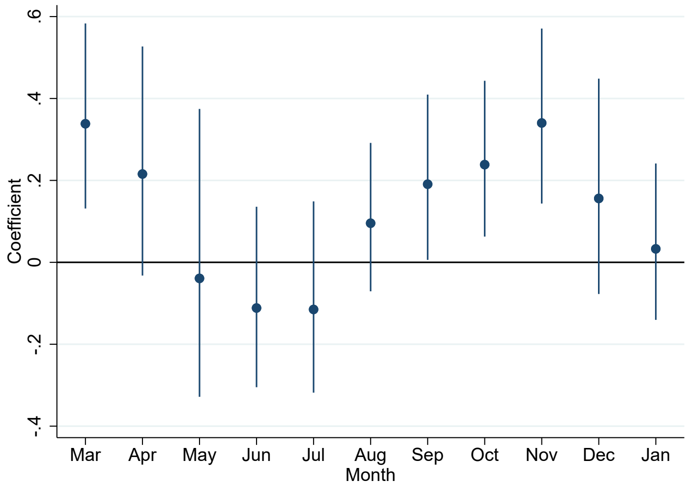

(a) Cases: Joint dummy (8, 9 or 10)

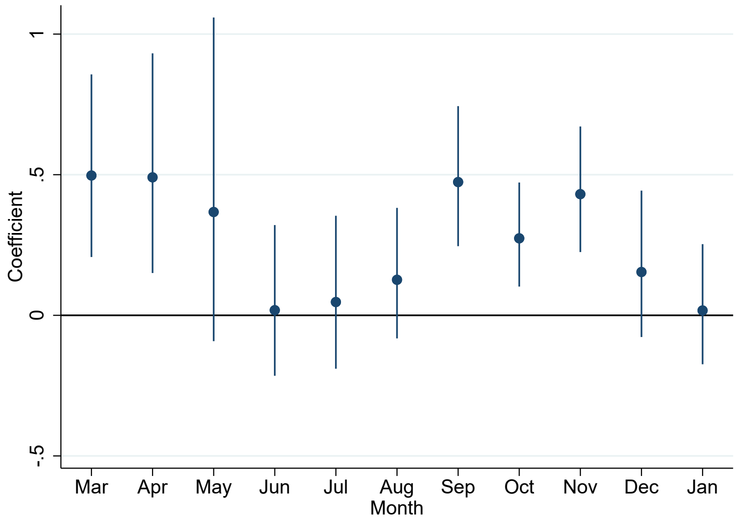

(b) Cases: Week 9 dummy

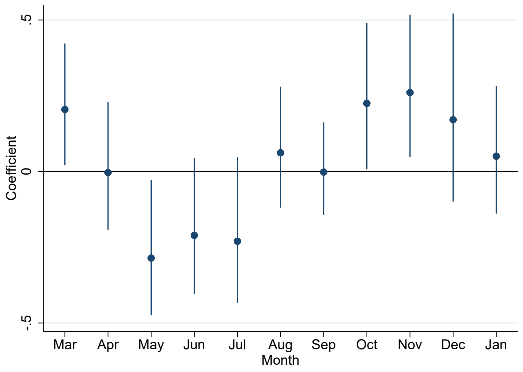

(c) Cases: Week 8 dummy

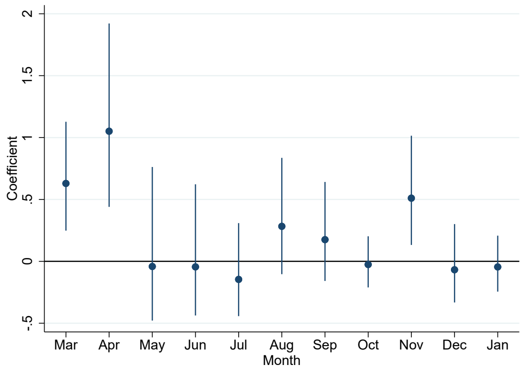

(d) Cases: Week 10 dummy

Figure F3: Gravity robustness: Coefficient plot of the joint dummy per month in graph F3a. Sub-graphs F3c,F3b F3d show week 8, 9 and 10 dummies (in a single regression without the joint dummy). Distance to Ischgl as a control variable

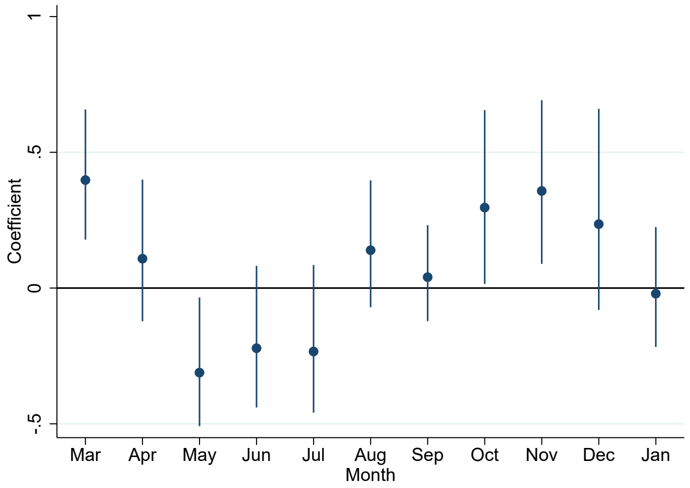

(a) Cases: Joint dummy (8, 9 or 10)

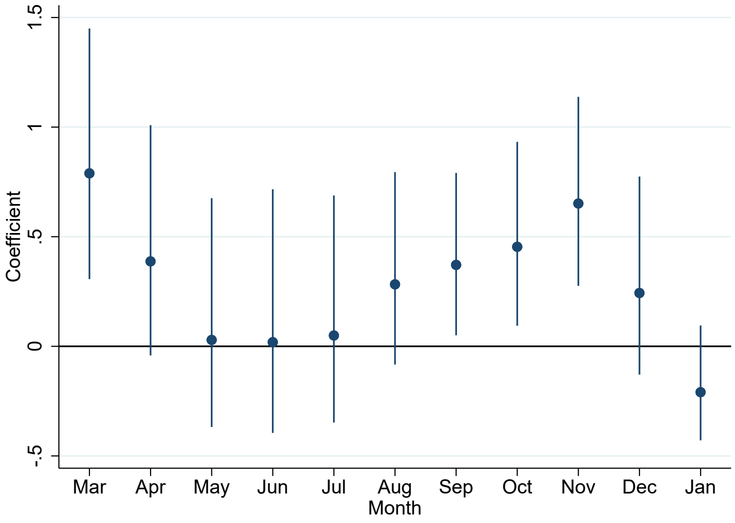

(b) Cases: Week 9 dummy

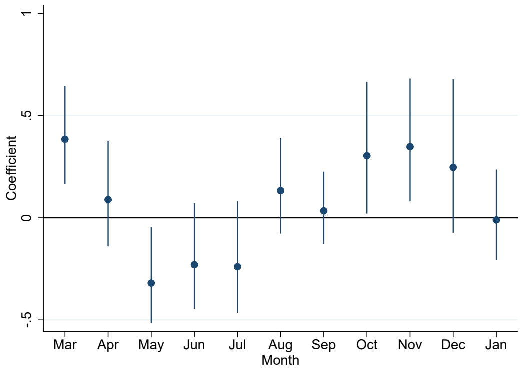

(c) Cases: Week 8 dummy

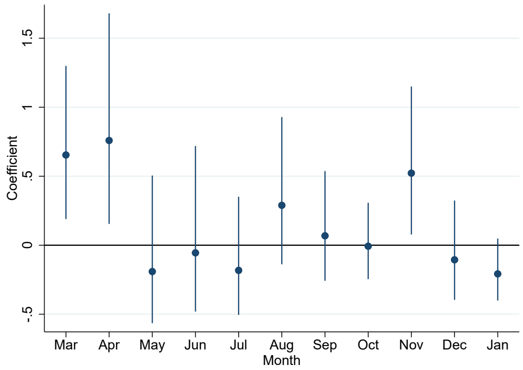

(d) Cases: Week 10 dummy

Figure F4: Gravity robustness: Dropping the two regions in Germany close to the Austrian Alps. Coefficient plot of the joint dummy per month in graph F4a. Sub-graphs F4c,F4b F4d show week 8, 9 and 10 dummies (in a single regression without the joint dummy). Full results available on request.
